# Stromal Cells Serve Drug Resistance for Multiple Myeloma via Mitochondrial Transfer: A Study on Primary Myeloma and Stromal Cells

**DOI:** 10.3390/cancers13143461

**Published:** 2021-07-10

**Authors:** Zsolt Matula, Gábor Mikala, Szilvia Lukácsi, János Matkó, Tamás Kovács, Éva Monostori, Ferenc Uher, István Vályi-Nagy

**Affiliations:** 1 Central Hospital of Southern Pest, National Institute of Hematology and Infectious Diseases, 1097 Budapest, Hungary; gmikala@dpckorhaz.hu (G.M.); uher.ferenc@gmail.com (F.U.); drvnistvan@gmail.com (I.V.-N.); 2 MTA-ELTE Immunology Research Group, Department of Immunology, Eötvös Loránd University, 1053 Budapest, Hungary; lukacsisz.zs@gmail.com; 3 Department of Immunology, Eötvös Loránd University, 1053 Budapest, Hungary; matko@elte.hu; 4 Department of Anatomy, Histology and Embryology, Semmelweis University, 1085 Budapest, Hungary; kovacs.tamas@med.semmelweis-univ.hu; 5 Institute of Genetics, Biological Research Centre, 6726 Szeged, Hungary; monosbeni@gmail.com

**Keywords:** multiple myeloma, tunneling nanotube, mitochondrial transfer, cancer drug resistance, bone marrow mesenchymal stromal cell

## Abstract

**Simple Summary:**

Mitochondrial transfer plays a crucial role in the acquisition of drug resistance in multiple myeloma, but its exact mechanism is not yet clear; moreover, overcoming the drug resistance that it causes is also a major challenge. Our research on primary myeloma cell cultures reveals that mitochondrial transfer is bi-directional between bone marrow stromal cells and myeloma cells, occurring via tunneling nanotubes and partial cell fusion with extreme increases under the influence of chemotherapeutic drugs, whereupon survival and adenosine triphosphate levels increase, while mitochondrial superoxide levels decrease in myeloma cells. These changes and the elevation of superoxide levels in stromal cells are proportional to the amount of incorporated mitochondria derived from the other cell type and to the concentration of the used drug. Although the inhibition of mitochondrial transfer is limited between stromal and myeloma cells, the supportive effect of stromal cells can be effectively averted by influencing the tumor metabolism with an inhibitor of oxidative phosphorylation in addition to chemotherapeutics.

**Abstract:**

Recently, it has become evident that mitochondrial transfer (MT) plays a crucial role in the acquisition of cancer drug resistance in many hematologic malignancies; however, for multiple myeloma, there is a need to generate novel data to better understand this mechanism. Here, we show that primary myeloma cells (MMs) respond to an increasing concentration of chemotherapeutic drugs with an increase in the acquisition of mitochondria from autologous bone marrow stromal cells (BM-MSCs), whereupon survival and adenosine triphosphate levels of MMs increase, while the mitochondrial superoxide levels decrease in MMs. These changes are proportional to the amount of incorporated BM-MSC-derived mitochondria and to the concentration of the used drug, but seem independent from the type and mechanism of action of chemotherapeutics. In parallel, BM-MSCs also incorporate an increasing amount of MM cell-derived mitochondria accompanied by an elevation of superoxide levels. Using the therapeutic antibodies Daratumumab, Isatuximab, or Elotuzumab, no similar effect was observed regarding the MT. Our research shows that MT occurs via tunneling nanotubes and partial cell fusion with extreme increases under the influence of chemotherapeutic drugs, but its inhibition is limited. However, the supportive effect of stromal cells can be effectively avoided by influencing the metabolism of myeloma cells with the concomitant use of chemotherapeutic agents and an inhibitor of oxidative phosphorylation.

## 1. Introduction

Understanding the molecular mechanisms of cancer drug resistance is critical in order to accomplish effective and long-lasting cancer treatment. The ‘intrinsic’ mechanisms of drug resistance include many cellular processes such as DNA damage repair, genomic instability, apoptosis inhibition, metabolic adaptation, and the activity of drug transporters [1]. In addition, recently it has also become clear that cellular interactions within the tumor microenvironment play at least as important a role in tumor progression and resistance to therapy as the intracellular mechanisms. The most essential mediators of intercellular signaling are soluble factors, extracellular vesicles, and tunneling nanotubes (TNTs) [2], furthermore, the interactions of membrane proteins followed by the exchange of large plasma membrane fragments, also known as trogocytosis, are also decisive in the communication between mesenchymal stromal cells (MSCs) and cancer cells [3]. All of these different types of interactions involve functional interference and the mutual acquisition of new cellular properties.

Tunneling nanotubes are long-distance intercellular connections that allow the exchange of various cargos between cells, from ions and small molecules to functional organelles such as mitochondria [4]. The horizontal mitochondrial transfer is of great importance, as the acquisition of cancer drug resistance seems to be strongly associated with TNT-mediated mitochondrial transfer. Therefore, mitochondria emerged as a crucial therapeutic target in cancer and in other common pathologies as well, such as heart attack, Parkinson disease, Alzheimer disease, fatty liver disease, muscular dystrophies, and even colitis [5]. A recent approach optimizing cancer therapy is targeting different cellular organelles, including the mitochondria [6]. Over the past decade, several studies have found that the acquisition of mitochondria from neighboring healthy cells and/or the transfer of damaged mitochondria to the healthy cells through TNTs increases the growth potential of tumor cells, provides survival benefits, enhances their chemoresistance, and certainly alters the metabolism and functional properties of the recipient tumor cells [7,8,9,10,11]. The acquisition of mitochondria via TNTs does not only increase the oxidative phosphorylation (OXPHOS) activity and adenosine triphosphate (ATP) level of tumor cells, but also indirectly affects their general metabolism, improves their proliferative and migratory properties, and increases the feasibility of developing resistance to chemotherapeutic treatment [12,13,14]. Tumor cell metabolism is characterized by an energy-saving mode ensured by high glycolytic activity and none, or decreased, OXPHOS [15], even in the presence of abundant oxygen. However, recent studies have shown that OXPHOS increased in certain tumor types [16,17,18]. Mitochondria have a major contribution in the generation of reactive oxygen species (ROS): electrons are released due to OXPHOS activity and molecular oxygen interacting with these electrons producing ROS. Oxidative stress induces a sharp decrease of mitochondrial membrane potential and facilitates the induction of the mitochondrial permeability transition pores (MPTPs). As a result of opening the MPTPs, antioxidant molecules such as glutathione are released from mitochondria, which reduce the ability of ROS neutralization. In addition, more free radicals are produced due to the loss of mitochondrial electron transport chain components through the MPTPs. Mitochondrial permeability transition is a central coordinating event of apoptosis, and thus targeting MPTP might be a propitious anticancer therapy due to the specificity and less chance of developing resistance mechanisms [19].

Mesenchymal stromal cells protect leukemic cells such as T cell acute lymphoblastic leukemia (T-ALL) [10] and acute myeloid leukemia (AML) [7,8] from chemotherapy; however, the exact mechanism of this protection has not been clearly revealed. In the case of T-ALL, the survival of leukemic cells upon treatment with drugs (cytarabine or mitoxantrone) was attributed to mitochondrial transfer toward stromal cells through TNTs. The stromal cells showed an increasing number of leukemic cell-derived mitochondria, while in parallel the intracellular levels of mitochondrial ROS and apoptotic rates were significantly reduced in both Jurkat and primary ALL cells [10]. By inhibiting the formation of membrane nanotubes with cytochalasin D, the chemoresistance of leukemic cells was radically reduced.

In the case of acute myeloid leukemia, superoxide generated by the NADPH oxidase 2 enzyme complex induces bone marrow stromal cells to deliver mitochondria to AML blasts through tumor cell-derived membrane nanotubes [7]. In AML cells co-cultured with mesenchymal stromal cells, mitochondrial mass and mitochondrial ATP production increased by 14% and up to 1.5-fold, respectively, and AML showed a higher survival rate upon drug treatment [8].

Additionally, B-cell precursor acute lymphoblastic leukemia (BCP-ALL) cells induced MSCs to produce pro-survival cytokines and chemokines such as interferon-γ-inducible protein 10, interleukin 8, and monocyte chemotactic protein-1 [20]. The precursor B cell-derived membrane nanotubes carry autophagosomes, mitochondria, endoplasmic reticulum, and the ICAM-1 transmembrane protein into mesenchymal stromal cells [9]. Bone marrow stromal cells can also transfer mitochondria through TNTs into B-ALL cells and rescue them from ROS-inducing chemotherapy [21], suggesting that mitochondrial transfer between BCP-ALL cells and MSCs can occur in both directions.

At present, only one study has been conducted on this topic in relation to multiple myeloma claiming that bone marrow stromal cells increased OXPHOS activity in MMs by the accumulation of functional mitochondria from BM-MSCs via TNTs, and CD38 was involved in this process [11].

In this study, primary myeloma cells (MMs) and autologous BM-MSCs were used. The purpose of the work was to obtain a deeper insight into the mechanism by which MSCs protect MMs from cytotoxic effect of chemotherapeutic drugs and therapeutical antibodies used in the treatment of multiple myeloma. In the course of testing these medicines, we determined the direction and kinetics of mitochondrial transfer. A variety of inhibitors blocking different cellular processes such as endocytosis, the formation of gap junctions, actin, and tubulin polymerization, and macropinocytosis were also used. Changes in mitochondrial specific superoxide and ATP levels in both donor and recipient cells were determined in parallel with the quantification of intercellular mitochondrial transfer in the presence or absence of drugs or inhibitors. The effect of the OXPHOS inhibitor metformin in combination with chemotherapeutic agents was also assessed regarding the survival of MMs in the presence of autologous BM-MSCs.

## 2. Materials and Methods

### 2.1. Primary Cell Isolation and Culture

All experiments using primary human cells were approved by the Ethics and Scientific Committee of the Central Hospital of Southern Pest—National Institute of Hematology and Infectious Diseases (OGYÉI/50268-8/2017). Bone marrow aspirates collected for diagnostic and research purposes were obtained by sternal bone marrow puncture after patients’ written informed consent. A list of involved patients is shown in Table 1. Bone marrow mononuclear cells (BM-MNCs) were isolated by density gradient centrifugation on Ficoll-Paque PLUS (GE Healthcare Bio-Sciences, Pittsburgh, PA, USA) according to the manufacturer’s instructions. The isolated BM-MNCs were cultured in DMEM/F12 growth medium supplemented by 10% *v*/*v* FBS, 2 mM L-glutamine, 100 IU/mL of Penicillin, and 100 µg/mL of Streptomycin. Medium and supplements were purchased from Thermo Fisher Scientific (Waltham, MA, USA). Culture medium was changed after 3 days, and primary cells were further cultured for up to 1–2 months, while the growth medium was changed twice a week. When the cell cultures contained only stromal cells and intensively proliferating malignant plasma cells, the co-cultures were passaged. One part of the cells was cryopreserved, while the other part was further cultured for the experiments as follows: proliferating myeloma cells were washed thoroughly from the stromal cell layer and cultured in a separate flask. The washing step was repeated a further two times, and the separation of the two cell types was improved by a repeated passage if needed, after which malignant plasma cells were washed thoroughly again from the newly adhered stromal cells. If the two cell types could not be separated this way, stromal cells were sorted by FACSAria flow cytometer (BD Biosciences, Franklin Lakes, NJ, USA). Only successfully separated cell cultures were used for further experiments, where the culture of stromal cells contained little or no myeloma cells. All primary bone marrow samples were obtained from patients with intramedullary myeloma.

### 2.2. Therapeutic Antibodies, Chemotherapeutic Drugs, and Inhibitors Used in the Cytotoxicity Assay and Mitochondrial Transfer Assays

The list of therapeutic antibodies and chemotherapeutic agents and inhibitors of various cellular processes used in cytotoxicity assays and mitochondrial transfer assays are detailed in Table 2.

### 2.3. In Vitro Cytotoxicity Assay

Toxic effects of various drugs on BM-MSCs or MM cell monocultures, or their co-cultures, were determined with the high-content screening (HCS) method. Briefly, a 1 × 10^3^ MSC/well and a 1 × 10^4^ myeloma cell/well were seeded in the DMEM/F12 medium on 96-well plates in monocultures or co-cultures. Cells were incubated in the presence or absence of different drugs for 72 h at 37 °C. After incubation, Hoechst 33,342 dye was added to each well in a final concentration of 100 ng/mL for 1 h in order to distinguish between myeloma cells and stromal cells based on the size of the nucleus. Finally, 1 µg/mL propidium iodide was added to each well. Results were evaluated using the CellReporterXpress Image Acquisition and Analysis Software (Molecular Devices, San Jose, CA, USA).

### 2.4. Mitochondrial Transfer Assay 

Bone marrow mesenchymal stromal cells or myeloma cells were stained with Mitotracker Red FM (Thermo Fisher Scientific) at a final concentration of 200 nM in 1X HBSS buffer at 37 °C for 15 min and then washed three times in 1X HBSS. BM-MSCs were seeded in a 24-well plate (Eppendorf, Hamburg, Germany) at a density of 2.5 × 10^4^ cells/well, while stained myeloma cells were seeded into T75 flasks (Eppendorf). Both cell types were further cultured for 72 h at 37 °C, then the stained cells were washed again three times with 1 x HBSS buffer. Co-cultures were established with 1:10 ratio of BM-MSCs (2.5 × 10^4^ cells/well): myeloma cells (2.5 × 10^5^ cells/well). MitoTracker-labeled BM-MSCs and unlabeled myeloma cells or MitoTracker-labeled myeloma cells and unlabeled BM-MSCs were seeded on 24-well plates and cells were incubated for different periods of time (2, 6, 12, 24, 48 h) with or without drug treatment (Supplementary Figure S1A). After co-culturing, the cells were trypsinized, washed, and incubated with fluorescently labeled monoclonal antibodies to distinguish myeloma cells from stromal cells. Monoclonal antibodies, anti-CD146 Alexa Fluor 488 and anti-CD38 Alexa Fluor 488 (both purchased from BioLegend, San Diego, CA, USA) were used to distinguish Mitotracker unlabeled BM-MSCs and myeloma cells, respectively, from those of Mitotracker labeled co-cultured cells. MitoTracker red fluorescence was analyzed by a FACSCanto II flow cytometer (BD Biosciences).

### 2.5. Transwell Assay 

Unlabeled BM-MSCs or MM cells were seeded into wells of 24-well plates (BD Biosciences) at a density of 2.5 × 10^4^ or 5 × 10^5^ cells/well, respectively, and paired with MitoTracker Red-stained myeloma cells or BM-MSCs seeded into the transwell inserts (BD Falcon, 353096, 3 µm pore size). After 1 or 2 days of co-culture in the presence or absence of chemotherapeutic drugs, unlabeled cells were measured for MitoTracker Red positivity by flow cytometry (Supplementary Figures S1B and S4).

### 2.6. Isolation of Microvesicles (MV) and Investigation of Their Role in Mitochondrial Delivery 

Bone marrow mesenchymal stromal cells and myeloma cells were stained with Mitotracker Red FM and washed, as described previously. Both cell types were further cultured for 72 h and then washed twice. Fetal bovine serum of the growth medium was replaced by KnockOut Serum Replacement (Thermo Fisher Scientific) in order to avoid contamination with extracellular vesicles present in FBS. For MV production, both cell types were grown into the log phase. Microvesicles were isolated by the combination of differential centrifugation and gravity-driven filtration as follows: After the collection of the culture medium, cells were removed by centrifugation at 300× *g* for 10 min, and thereafter, the cell debris was removed by 2000× *g* centrifugation for 10 min. The supernatants were filtered by gravity through a 0.8 µm syringe filter unit (Merck) to completely remove apoptotic bodies and centrifuged for 20 min at 12,500× *g* at room temperature (Supplementary Figure S1C). The concentration of MV isolates was determined by TRPS analysis using a qNano device (IZON Science, Christchurch, New Zealand), as described previously [2]. Calibration was achieved using CPC400 calibration beads (IZON Science, Christchurch, New Zealand). At least 500 events were registered with a linear particle rate in time using NP400 nanopore membrane (IZON Science) stretched between 45 and 47 mm. The voltage was set to 0.2–0.34 V to achieve a stable average current (126–130 nA) with a low average RMS noise. Microvesicles were stained with PKH67 membrane labeling dye (Merck). Microvesicle suspension derived from donor cells, the Mitotracker Red-stained BM-MSC or MM cells were cultured with recipient cells, unstained myeloma cells, or BM-MSCs, respectively, at 6 × 10^4^ MVs/cell ratios for 24 h. The percentage of PKH67 and Mitotracker Red FM positive recipient cells was determined by flow cytometry.

### 2.7. Determination of Mitochondrial Superoxide Levels

The mitochondrial superoxide levels in monocultures or co-cultures were detected using the MitoSOX Red fluorogenic reagent (Thermo Fisher Scientific). Monocultures or co-cultures were trypsinized, washed with HBSS, and incubated in the MitoSOX Red (Thermo Fisher Scientific) working solution (5 µM dye in HBSS) at 37 °C for 10 min. After washing the cells with prewarmed HBSS buffer thrice, cells were analyzed by flow cytometry.

### 2.8. Determination of Mitochondrial ATP Levels in Living Cells

Cells were plated in a monoculture or in an MM–BM-MSC co-culture for 24 h with or without drug treatment. After 24 h, MM cells or BM-MSCs were labeled with 10 µM of BioTracker ATP-Red dye (Merck) for 15 min at 37 °C then washed thrice with PBS. ATP level was analyzed in the samples with flow cytometry.

### 2.9. Confocal Laser Scanning Microscopy

For confocal laser scanning microscopy, µ-Slide eight-well glass bottom imaging chambers (Ibidi, Gräfelfing, Germany) were coated with 10 µg/cm^2^ human plasma fibronectin (Merck, Darmstadt, Germany). Bone marrow mesenchymal stromal cells or myeloma cells were stained with Mitotracker Red FM, as described previously. Myeloma cells were labeled with Vybrant DiI cell-labeling solution (Thermo Fisher Scientific) as follows: cells were washed with PBS and then incubated with the prewarmed dye solution (5 mg DiI/mL in PBS) for 5 min at 37 °C. After incubation, the cells were washed thrice with culture medium. Myeloma cells (1 × 10^5^ cells/cm^2^) were seeded on the imaging chambers and co-cultured with the stromal cells (1 × 10^4^ cells/cm^2^) for 24 h. Mitotracker stained BM-MSCs or MM cells were co-cultured with unstained myeloma cells (labeled with Vybrant DiI) or BM-MSCs (labeled anti-CD146 eFluor 450), respectively. The imaging chambers were incubated in a heating and incubation system (Ibidi) during the whole process at 37 °C with 5% CO_2_. Finally, the samples were examined with an Olympus FluoView 500 Laser Scanning Confocal Microscope (Shinjuku, Tokyo, Japan) with a 60× oil immersion objective and analyzed with FluoView application software (Ver. 05.00.110). For the experiments with fixed samples, co-cultures were fixed with 4% PFA solution for 10 min at room temperature. Prior to and after fixation, cells were washed with dPBS buffer thrice. These samples were examined with a Zeiss LSM 780 Laser Scanning Confocal Microscope (Zeiss, Oberkochen, Germany), and data were analyzed with Zen 3.3 (blue edition) application software.

### 2.10. Live Imaging of Mitochondrial Transfer in Co-Cultures with High-Content Screening Method

The process of mitochondrial transfer was investigated using time-lapse imaging using the ImageXpress Pico Automated Cell Imaging System (Molecular Devices). BM-MSCs were labeled with MitoTracker Red FM, as described previously, while myeloma cells were unlabeled. The number of mitotracker-positive myeloma cells was quantified using a 20× objective by analyzing 0.69 mm × 0.69 mm areas (0.4761 mm^2^). The images were taken from the beginning of the establishment of the co-cultures for 1 h with an interval of 8311 ms. The time-lapse video thus consisted of a total of 434 images.

### 2.11. Lentiviral Gene Transfer 

The lentiviral product identified as ‘Mitochondria Cyto-Tracer, pCT-Mito-GFP (CMV)’ was purchased from System Biosciences (Palo Alto, CA, USA). Primary myeloma cells and BM-stromal cells were transduced with lentiviral particles delivering sequences that expressed fluorescent protein tags (AcGFP1) targeted specifically to the mitochondria (Supplementary Figure S7). Both cell types were transduced with pCT-Mito-GFP at a multiplicity of infection (MOI) of approximately 10 (BM-MSC) and 25 (MM). GFP-positive stromal cells and myeloma cells were sorted by FACSAria flow cytometer (BD Biosciences).

### 2.12. Statistical Evaluation

The data are presented as the mean of three repeated experiments of biological parallels ± SD. Statistical differences were evaluated using Student’s *t*-test. *p* values < 0.05 were accepted as significant.

## 3. Results

### 3.1. Effect of BM-MSCs on MM Cells’ Survival in the Presence of Toxic Concentration of Various Drugs

**Figure 1:**
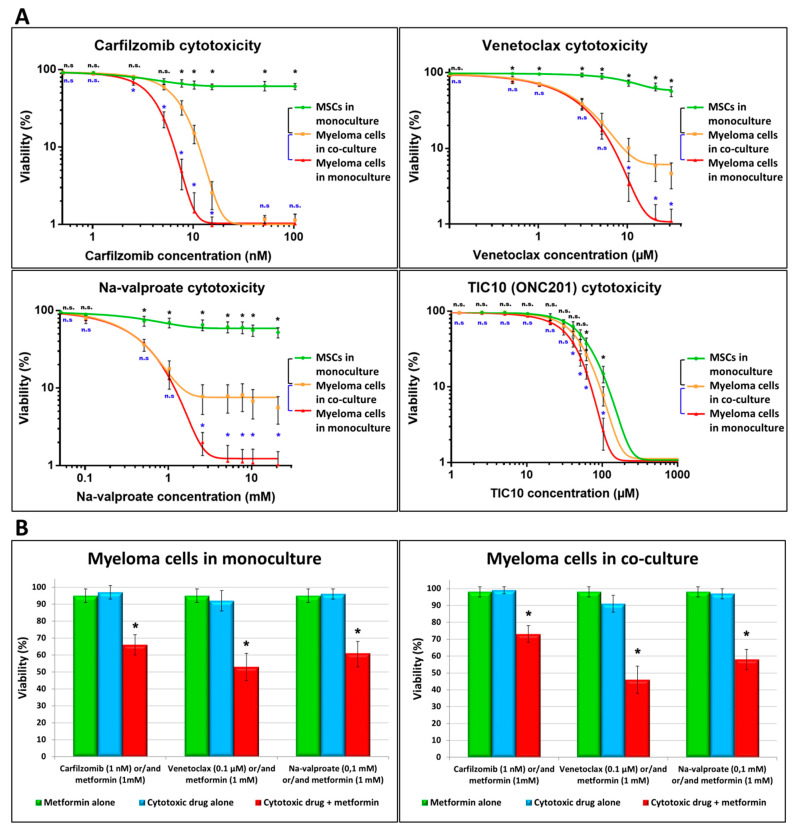
Effect of BM-MSCs on the cytotoxicity of drugs exerted on MMs. Cells (1 × 10^3^ BM-MSC/well, 1 × 10^4^ myeloma cell/well in monocultures or MM–BM-MSC co-cultures) were incubated for 72 h in the absence or presence of various drugs then labeled with Hoechst 33,342 and propidium iodide. Cell death was analyzed with a high-content screening method using an automated digital microscope system (A and B). (**A**) Cytotoxicity of different concentrations carfilzomib, venetoclax, Na-valproate, and TIC10 was determined in MMs (red line) and BM-MSC (green line) monocultures or MM–BM-MSC (orange line) co-cultures. (**B**) The drugs (blue columns) carfilzomib (1nM), venetoclax (0.1 µM), Na-valproate (0.1 mM), and metformin (1 mM, green column) were added to the MM monoculture (left graph) or MM–BM-MSC co-culture (right graph) alone or in combination (red column). These results are representative of three independent experiments for one patient. The values are presented as mean ± standard deviation, *p* values < 0.05 were considered significant (*) while *p* values > 0.05 were considered non-significant (n.s.).

To determine the cytotoxicity of different drugs on primary myeloma cell cultures, we examined the effect of various drugs on the viability of myeloma cells and bone marrow stromal cells both in monocultures and BM-MSC–MM co-cultures (Figure 1A). Cell cultures were treated with the proteasome inhibitor carfilzomib (0–100 nM), the BCL-2 inhibitor venetoclax (0–13 μM), the histone deacetylase inhibitor sodium-valproate (0–11 mM), and the mitochondrion-damaging TIC10 (0–100 μM) at various drug concentrations.

The low concentrations of the drugs carfilzomib (1 nM), venetolax (0.1 μM), Na-valproate (0.1 mM), and TIC10 (10 μM) were not toxic to either MMs or BM-MSCs. The presence of higher concentrations of any of the drugs resulted in a steep decrease in the viability of MMs, and the highest concentrations, 100 nM of carfilzomib, 30 μM of venetolax, 20 mM of Na-valproate, and 100 μM of TIC10) left no cells alive in the MM cultures. On the contrary, BM-MSCs were resistant to the drugs even in the highest concentrations, except TIC10, which showed similar toxicity to BM-MSCs as it did to MMs. BM-MSCs affected drug-induced death of the MMs. In the co-culture, BM-MSCs efficiently protected MMs from apoptosis caused by carfilzomib and a high concentration of the drugs venetoclax and Na-valproate. Although large differences in cytotoxicity were observed between co-cultures derived from different donors, the following trend was evident for all cell cultures: in the presence of carfilzomib, BM-MSCs effectively protected MM cells from apoptosis at all concentrations toxic to the MM cells. In contrast, in the presence of venetoclax and na-valproate, this protective effect occurred only at high drug concentrations. As TIC10 inhibited mitochondrial functions of both MMs and BM-MSCs, this drug had little effect on MM survival. Therefore, this drug was omitted from the further experiments.

Recently, antibodies have been introduced as biological treatments in MM therapy [23,24,25,26,27,28,31]. The therapeutical antibodies Daratumumab (CD38), Isatuximab (CD38), and Elotuzumab (CD139) were tested regarding their cytotoxic effects on MMs in concentrations found in the sera of patients found after therapy [32]. None of these antibodies were toxic to MMs or BM-MSCs in monocultures or co-cultures (Supplementary Figure S2).

Pharmacological approaches shifting the metabolism of leukemic cells toward lower oxidative phosphorylation significantly enhance the effect of anti-leukemic drugs [13]. Therefore, we tested the cytotoxic effect of metformin, an agent inhibiting oxidative phosphorylation [13,30], in combination with carfilzomib, venetoclax, and Na-valproate, on MM monocultures or BM-MSC–MM co-cultures. As shown in Figure 1B, even the non-toxic dose of metformin significantly reduced the viability of MMs in both monocultures and co-cultures when used together with a non-toxic dose of chemotherapeutic drugs (carfilzomib, venetoclax, Na-valproate). BM-MSCs in co-cultures did not affect appreciably MMs’ survival in the presence of these drug combinations despite the escalating bidirectional MT.

### 3.2. Mitochondrial Transfer between BM-Mscs and Mms in the Presence of Chemotherapeutic Drugs and Therapeutic Antibodies

**Figure 2:**
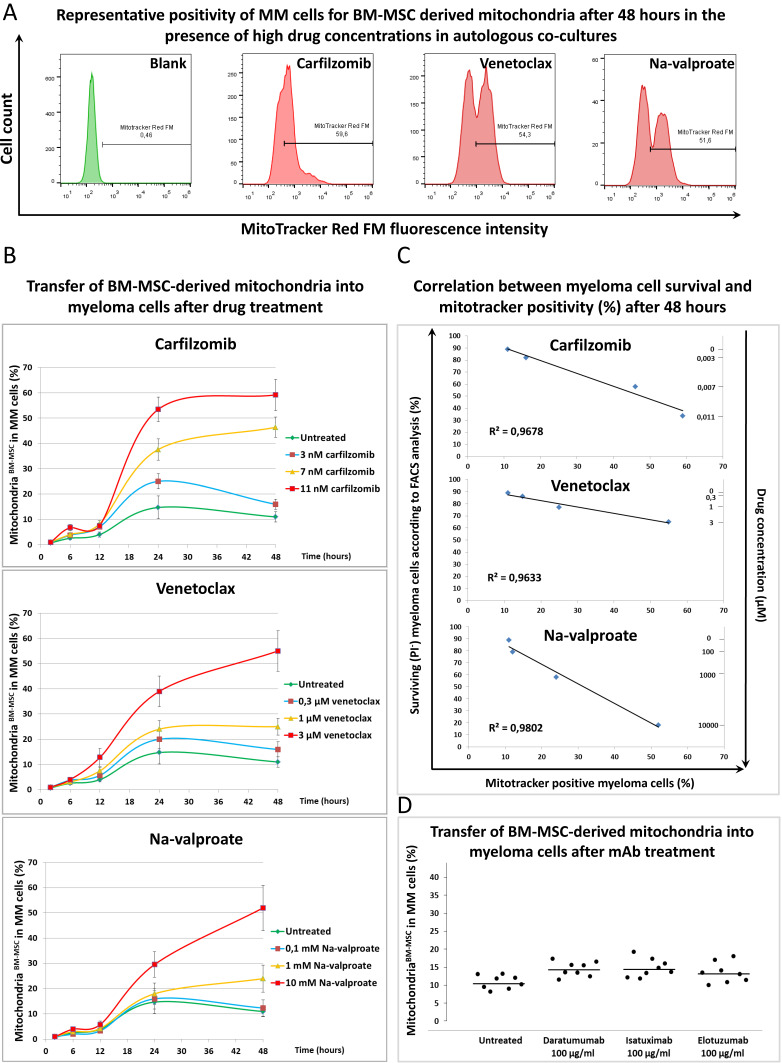
Mitochondrial transfer from bone marrow stromal cells to myeloma cells in the presence of chemotherapeutic drugs and therapeutic antibodies. (**A**) BM-MSCs were labeled with Mitotracker Red FM then co-cultured with MMs for 48 h in the presence or absence of the highest concentrations of each drug: 3 µM of venetoclax, 10 mM of Na-valproate, and 11 nM of carfilzomib. BM-MSC-derived mitochondria^+^ MMs were analyzed by flow cytometry within the CD38^+^ myeloma cell population. (**B**) Unlabeled MMs and Mitotracker Red FM-labeled BM-MSCs were co-cultured for 2, 6, 12, 24, and 48 h in the presence or absence of carfilzomib (3, 7, or 11 nM), venetoclax (0.3, 1, or 3 μM), Na-valproate (0.1, 1, or 10 mM), and mitochondrial transfer from BM-MSCs to MMs was analyzed as described under (**A**). (**C**) Correlation between survival of MMs and content of BM-MSC-derived mitochondria in the surviving MMs was evaluated in the presence or absence of different drug concentrations: 3, 7, or 11 nM of carfilzomib; 0.3, 1, or 3 μM of venetoclax; 0.1, 1, or 10 mM of Na-valproate. (**D**) The effect of therapeutic antibodies on mitochondrial transfer from BM-MSCs to MMs after 48 h of co-culture was analyzed as described under (**A**). The results of (**A**–**C**) panels are representative of three independent experiments for one patient. Panel (**D**) represents the averages of three independent experiments for 8 patients.

To understand the mechanism by which BM-MSCs prevent MMs from drug-induced cytotoxicity, mitochondrial transfer (MT) was followed between BM-MSCs and MMs in the presence or absence of various cytotoxic drugs at different time points (Figure 2). BM-MSC-derived mitochondria (labeled with Mitotracker Red FM dye) transferred into MMs (gated using CD38 antibody) even without any drug treatment, an average of 8–15% of MMs were positive for BM-MSC-derived mitochondria after 48 h of co-culture. The gating strategy for FACS analysis of mitochondrial transfer is shown in Supplementary Figure S8. All drugs increased the uptake of BM-MSC-derived mitochondria by MMs. The time course of the MT in the presence or absence of various drug concentrations showed that no transfer was observed in up to 12 h of the co-culture (Figure 2B). Between 12 and 24 h, MT sharply increased when using the highest concentration of the drugs. Then, up to 48 h, the presence of the highest concentrations of the drugs affected the transfer of BM-MSC-derived mitochondria into MMs differently. MT, stimulated by 11 nM of the proteasome inhibitor carfilzomib, reached a plateau or a very slight increase, respectively, while it was elevated in the presence of 3 μM of venetoclax (BCL-2 inhibitor) or 10 mM of Na-valproate (histone deacetylase inhibitor).

The effects of lower concentrations of the drugs showed concentration dependence regarding MT from BM-MSCs to MMs (Figure 2B). The positivity of MMs for BM-MSC-derived mitochondria in the presence of the highest drug concentrations after 48 h of co-culture is shown in Figure 2A. An unambiguous correlation was found between the survival of MMs, the amount of drugs added, and the BM-MSC-derived mitochondrial incorporation of the surviving myeloma cells. An increased amount of various drugs induced increased cell death of MMs; however, the surviving cells incorporated an increasing amount of BM-MSC-derived mitochondria (Figure 2C). These results suggest that BM-MSC-derived mitochondria served as a survival signal for the malignant plasma cells, and hence MMs were more resistant to the cytotoxic effect of the drugs used. Therapeutic antibodies did not exert a cytotoxic effect on MMs (Supplementary Figure S2), and, as expected, these non-toxic monoclonal antibodies did not attenuate the transfer of BM-MSC-derived mitochondria to MMs (Figure 2D).

**Figure 3:**
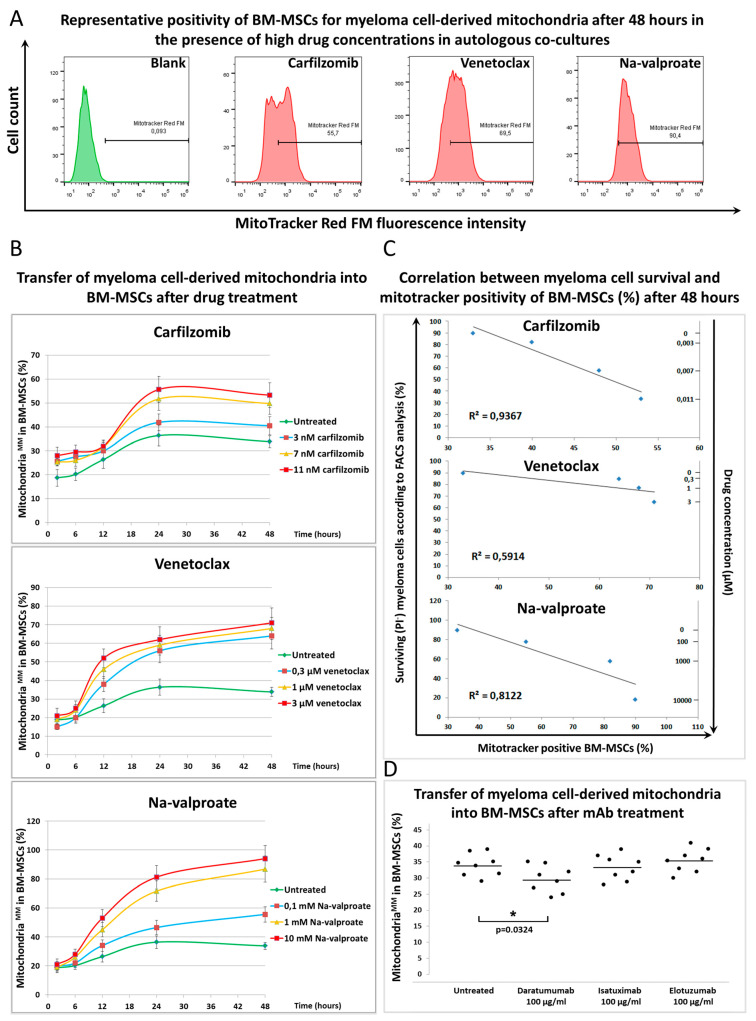
Mitochondrial transfer from myeloma cells to bone marrow stromal cells in the presence of chemotherapeutic drugs and therapeutic antibodies. (**A**) MMs were labeled with Mitotracker Red FM then co-cultured with BM-MSCs for 48 h in the presence or absence of the highest concentrations of each drug: 3 µM of venetoclax, 10 mM of Na-valproate, and 11 nM of carfilzomib. MM cell-derived mitochondria^+^ BM-MSCs were analyzed by flow cytometry within the CD146^+^ BM-MSC population. (**B**) Mitotracker Red FM-labeled MMs and BM-MSCs were co-cultured for 2, 6, 12, 24, and 48 h in the presence or absence of carfilzomib (3, 7, or 11 nM), venetoclax (0.3, 1, or 3 μM), Na-valproate (0.1, 1, or 10 mM), and mitochondrial transfer from BM-MSCs to MMs was analyzed as described under (**A**). (**C**) The correlation between MM cell survival and content of MM cell-derived mitochondria in BM-MSCs was evaluated in the presence or absence of different drug concentrations: 3, 7, or 11 nM carfilzomib; 0.3, 1, or 3 μM venetoclax; 0.1, 1, or 10 mM Na-valproate. (**D**) The effect of therapeutic antibodies on mitochondrial transfer from MMs to BM-MSCs after 48 h of co-culture was analyzed as described under (**A**). The results of (**A**–**C**) panels are representative of three independent experiments for one patient. Panel (**D**) represents the averages of three independent experiments for 8 patients. The values are presented as mean ± standard deviation, *p* values < 0.05 were considered significant (*).

To test whether the unidirectional transfer of mitochondria from BM-MSCs to MMs or bidirectional exchange occurred between the two cell types, MT from MMs to BM-MSCs were examined by analyzing MM cell-derived mitochondria in CD146^+^ BM-MSCs after 48 h of co-cultures in the presence of various drugs. The gating strategy for FACS analysis of mitochondrial transfer is shown in Supplementary Figure S8. A large amount (55–90% of recipient cells were positive) of MM cell-derived mitochondria was detected in BM-MSCs (Figure 3A) depending on the drug used. On the other hand, MT from MMs to BM-MSCs was detected as early as 2 h after treatment of the co-cultures with drugs since 15–30% of BM-MSCs became positive for MM cell-derived mitochondria (Figure 3B). Note that MT in the opposite direction (from BM-MSCs to MMs) remained under 5% at this early time point (Figure 2B). The early (2 h of co-culture) incorporation of mitochondria by BM-MSCs indicated that this transfer mechanism may occur via endocytosis of MMs undergoing an early apoptotic phase induced by drug cytotoxicity. This assumption was supported by the finding that 60% of the BM-MSCs were able to phagocytose apoptotic tumor cells as early as 3 h after induction of apoptosis [33].

Between 6 and 12 h, the stromal cells showed a continuous elevation of incorporation of MM cell-derived mitochondria in the presence of any concentrations of all drugs and then reached a plateau for up to 48 h of co-culture in a drug dose-dependent fashion (Figure 3B). A strong correlation was found between the mortality of MMs (drug cytotoxicity), MM cell-derived mitochondrial incorporation by the BM-MSCs, and the used drug concentrations. The more MMs died upon treatment with increasing amounts of drugs, the more MM cell-derived mitochondria appeared in BM-MSCs (Figure 3C). Therapeutic antibodies did not inhibit MT from BM-MSC to MMs (Figure 2D) except Daratumumab, which significantly decreased the amount of transferred mitochondria from MMs to BM-MSCs (Figure 3D).

### 3.3. Mitochondrial Transfer in Transwell Experiments and the Role of Microvesicles in the Intercellular Mitochondrial Transfer

Mitochondrial transfer between different cells may occur by direct cell-to-cell contact or by endocytosis of mitochondria containing vesicles. To determine the mechanism of MT, the mitochondrial transfer assays were performed in transwell chambers. Unlabeled recipient cells (BM-MSCs or MMs) were seeded into the bottom of 24-well plates, while MitoTracker-stained donor cells (MMs or BM-MSCs, respectively) were seeded into the transwell inserts (Supplementary Figure S1). No MT was detected in either direction after 2 days in transwell cultures, indicating that MT required direct cell–cell contact (Supplementary Figure S3).

However, when microvesicles (MVs) were isolated from the supernatant of MitoTracker-labeled BM-MSCs or MMs and incubated with unlabeled MMs or BM-MSCs (5 × 10^4^ MV/cell on average), respectively, different results were obtained. The isolated MVs were always labeled with the PKH67 membrane labeling kit before incubating with the unlabeled recipient cells in order to determine whether the recipient cells are able to incorporate the donor cell-derived MVs or not. After 24 or 48 h of co-culture, MMs did not accumulate considerable amounts of membrane components from PKH67-labeled MVs derived from BM-MSCs, and, accordingly, MMs did not accumulate BM-MSC-derived mitochondria (Supplementary Figure S4A). In contrast, after the fluorescently labeled MV treatment, most of the acceptor BM-MSCs was highly positive for the fluorescent membrane labeling dye and also for MM cell-derived mitochondria indicating that BM-MSCs are capable of incorporating microvesicles released from MMs (Supplementary Figure S4B).

In summary, MVs have no role in horizontal mitochondrial transfer using physiological cell/MV ratios, as shown in the transwell experiments. Although MMs are unable to incorporate MVs of BM-MSC origin even when incubated with large amounts of MVs (5 × 10^4^ MV/cell on average), stromal cells are unquestionably able to incorporate mitochondria-containing MVs released by MMs when incubated with concentrated MVs.

### 3.4. Determination of Mitochondrial Superoxide Levels

**Figure 4:**
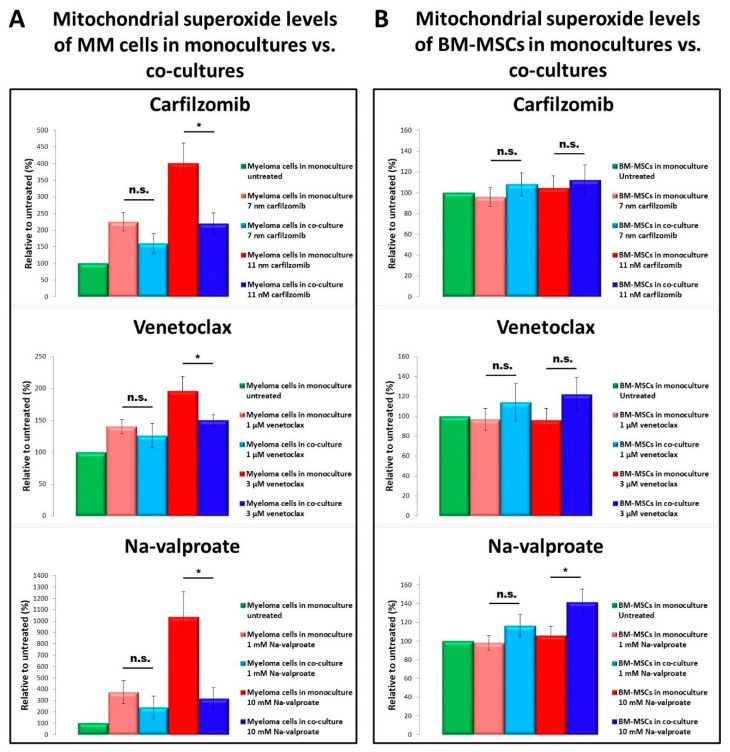
Mitochondrial superoxide levels in MMs and BM-MSCs. The cells were treated with various drugs in mono- or co-cultures and cultured for 24 h (**A**,**B**). After washing, all cells of the co-cultures were labeled with MitoSOX Red for 10 min at 37 °C. After subsequent washing, superoxide levels were analyzed by flow cytometry within the CD38^+^ MM cell population (**A**) or within the CD146^+^ BM-MSC population (**B**) using anti-CD38 Alexa Fluor 488 or anti-CD146 Alexa Fluor 488 monoclonal antibodies, respectively. The values are presented as mean ± standard deviation, *p* values < 0.05 were considered significant (*) while *p* values > 0.05 were considered non-significant (n.s.). The results are representative of three independent experiments for one patient.

One of the effects of cytotoxic drugs on myeloma cells is triggering the production of free radicals in their mitochondria. Indeed, all used drugs induced an increase in free radicals in MMs in monocultures (Figure 4A). When we used a higher concentration of the drugs, the presence of BM-MSCs in co-cultures significantly decreased the amount of free radicals in MMs (Figure 4A).

In contrast, none of the treatment of BM-MSCs with drugs resulted in a free radical increase in BM-MSC monocultures, while in co-cultures with MMs, free radical levels increased significantly in the presence of Na-valproate and non-significantly in the presence of carfilzomib or venetoclax (Figure 4B). The increase in free radical levels in BM-MSCs in co-cultures was explained by the incorporation of MM cell-derived mitochondria by BM-MSCs.

### 3.5. Determination of ATP Levels

**Figure 5:**
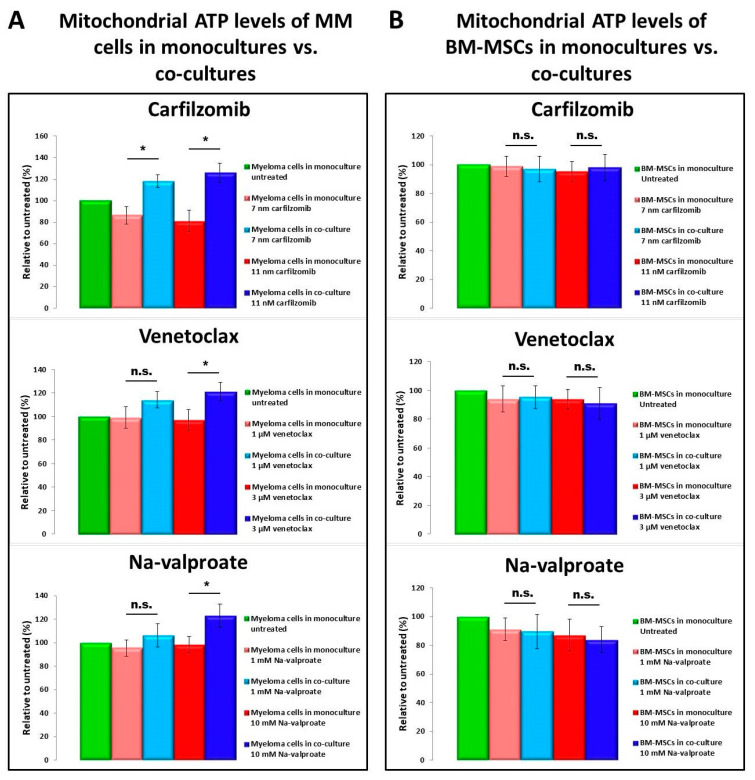
Mitochondrial ATP levels in MMs and BM-MSCs. BM-MSCs (**B**) or MMs (**A**) were plated in monocultures or in co-cultures and incubated for 24 h in the presence or absence of various drugs. After washing, cells were labeled with BioTracker ATP-Red for 15 min at 37 °C. Finally, after subsequent washing, ATP levels were analyzed by flow cytometry within the CD38^+^ MM cell population (**A**) or within the CD146^+^ BM-MSC population (**B**) using anti-CD38 Alexa Fluor 488 or anti-CD146 Alexa Fluor 488 monoclonal antibodies, respectively. The values are presented as mean ± standard deviation, *p* values < 0.05 were considered significant (*) while *p* values > 0.05 were considered non-significant (n.s.). The results are representative of three independent experiments for one patient.

Chemotherapeutic drugs, which affected cell survival, mitochondrial transfer, and mitochondrial functions, might also change energy, e.g., ATP production. In myeloma monocultures, mitochondrial ATP levels significantly decreased after carfilzomib treatment, while they showed little or no change upon venetoclax or Na-valproate treatment compared to untreated MMs (Figure 5A).

None of the drugs affected ATP production by BM-MSCs (Figure 5B) significantly, but the mitochondrial ATP level of stromal cells slightly decreased after drug treatment. Co-culturing MMs with BM-MSCs resulted in significant increase in mitochondrial ATP levels in MMs compared to the corresponding MM monocultures (Figure 5A), supporting the view, that ‘healthy’ BM-MSC-derived mitochondria serve as an ATP source for MMs. Contrarily, myeloma cells did not affect mitochondrial ATP levels of BM-MSCs in co-cultures in the presence of drugs (Figure 5B).

### 3.6. Confocal Laser Scanning Microscopy of BM-MSC–MM Co-Cultures

**Figure 6:**
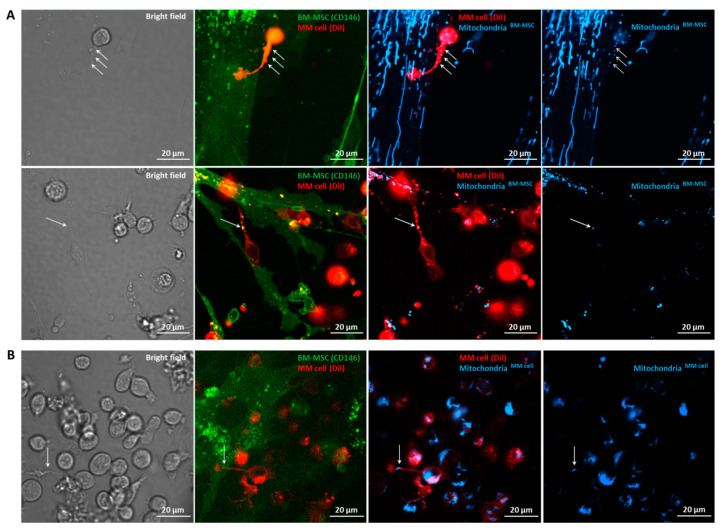
Visualization of tunneling nanotubes (TNTs) during mitochondrial transfer between BM-MSCs and MMs with confocal laser scanning microscopy. BM-MSCs and MMs were labeled with anti-CD146 conjugated with eFluor 450 (green) or with Vybrant DiI membrane-labeling dye (red), respectively (**A**,**B**). The mitochondria in BM-MSCs (**A**) or MMs (**B**) were stained with MitoTracker Red FM dye (blueish). MitoTracker Red FM-labeled BM-MSCs (**A**) or MMs (**B**) were co-cultured for 24 h with the recipient cells. A and B, left, show the bright field images of BM-MSC–MM co-cultures. The second column images show overlaps between BM-MSCs and MMs; the third column images show overlaps between BM-MSC-derived mitochondria in BM-MSCs and MMs (**A**) or MM cell-derived mitochondria in MMs and in BM-MSCs (**B**), and images on the right show BM-MSC-derived mitochondria (**A**) or MM cell-derived mitochondria (**B**). White arrows show the mitochondrial transfer from BM-MSCs to MMs (**A**) or from MMs to BM-MSCs (**B**) through MM cell-derived TNTs.

To clarify the mechanisms responsible for mitochondrial transfer between human primary BM-MSCs and myeloma cells, we analyzed the co-cultures with confocal laser scanning microscopy. Previous studies showed that transfer of mitochondria from non-malignant stromal cells into malignant cells and/or the transfer of damaged mitochondria to the stromal cells occurred via tunneling nanotubes [7,8,9,10,11,20]. Hence, we analyzed the role of tunneling nanotubes in MT between primary BM-MSCs and MMs. The stromal cells and myeloma cells were distinguished with anti-CD146 eFluor 450 conjugated antibody and Vybrant DiI membrane-labeling dye, respectively. The mitochondria of the donor cells were labeled with Mitotracker Red FM dye, and after 24 h of co-culture, the transfer of these organelles into the recipient cells was investigated (Figure 6). Mitochondrial transfer was visualized both from BM-MSC to MMs (Figure 6A) and from MMs to BM-MSC (Figure 6B) through MM cell-derived tunneling nanotubes indicated by white arrows; hence, we can clearly state that myeloma cell-derived TNTs play an important role in mitochondrial delivery in both directions. In contrast, no organelle transfer was detected in either direction via BM-MSC-derived TNTs.

### 3.7. Effects of Different Inhibitors on Mitochondrial Transfer

**Figure 7:**
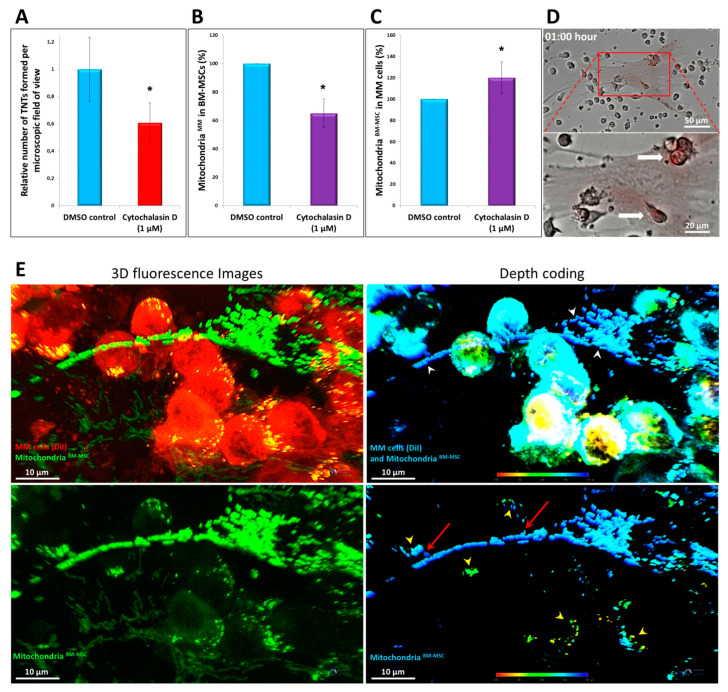
High-resolution 3D analysis of mitochondrial transfer using confocal laser scanning microscopy and the effect of cytochalasin D on mitochondrial transfer and TNT formation. BM-MSCs were labeled with MitoTracker Red FM (**A**–**D**); BM-MSCs were expressing mitochondrial-targeted AcGFP1 fluorescence protein (**E**). MMs were unlabeled (**B**,**C**) or were labeled with Vybrant DiI membrane-labeling dye (**A**,**E**). The cells were kept in co-cultures for 1 h (**D**) or 24 h (**A**–**C**,**E**) in the presence (**A**–**C**) or absence (**D**,**E**) of cytochalasin D. Mitochondrial transfer from BM-MSC to MMs was evaluated with a high-content screening method (**D**), flow cytometry (**B**,**C**), or confocal scanning microscopy (**A**,**E**), respectively. (**A**) BM-MSCs (labeled with MitoTracker Red FM) and MMs (labeled with Vybrant DiI) were co-cultured in the absence (DMSO control) or presence of 1 µM of cytochalasin D, and TNT quantitation was evaluated with confocal scanning microscopy counting the total number of TNTs between BM-MSCs and MMs per field of view using a 60x oil immersion objective. (**B**) Mitochondrial transfer from MMs to BM-MSCs was analyzed as described in Figure 3 in the presence or absence of cytochalasin D. (**C**) Mitochondrial transfer from BM-MSCs to MMs was analyzed as described in Figure 2 in the presence or absence of cytochalasin D. (**D**) Mitotracker-labeled BM-MSCs were co-cultured with MMs for 1 h and analyzed with time-lapse imaging with an automated digital microscope system. (**E**) 3D analysis of mitochondrial transfer between the stromal cells and MMs using confocal laser scanning microscopy (63x oil immersion objective). BM-MSCs were expressing mitochondrial-targeted AcGFP1 fluorescence protein, while MMs were labeled with Vybrant DiI membrane-labeling dye. After 24 h of co-culture in the presence of 6 nM carfilzomib (in order to increase MT), cells were fixed with 4% PFA solution for 10 min at room temperature. White arrowheads indicate the Ac-GFP1-tagged mitochondria of BM-MSCs; yellow arrowheads indicate the BM-MSC-derived mitochondria inside the MMs (double positive in the fluorescence image). Red arrows indicate BM-MSC-derived mitochondria transferring into the MMs. The values are presented as the mean ± standard deviation, and *p* values < 0.05 were considered significant (*) ((**A**–**C**): results are representative of three independent experiments for 1 patient).

According to the literature, the exclusive role of TNTs in mediating bidirectional mitochondrial transfer has not yet been clearly demonstrated. Therefore, cytochalasin D, an inhibitor of actin polymerization, was used to block TNT formation between the stromal cells and MMs. Cytochalasin D significantly inhibited mitochondrial transfer from MMs to BM-MSCs by an average of 35% (Figure 7B) and also significantly impeded TNT formation since it reduced the number of TNTs linking BM-MSCs and myeloma cells by up to 40% on average (Figure 7A). However, mitochondrial delivery from BM-MSCs to MMs was not affected by this compound. Consequently, cytochalasin D was shown to inhibit TNTs, but, importantly, while it inhibited MT from MMs to BM-MSC, it did not block MT from BM-MSC to MMs (Figure 7C).

These results indicated that MT may differ in its mechanism depending on its direction. Therefore, other inhibitors, colcemid, dynasore, EIPA, 18-α-GA, defibrotide, and metformin, affecting cell shape, cell–cell interaction junctions, microvesicle endocytosis, macropinocytosis, and cell metabolism, were used to analyze the mechanism of MT in one direction (from BM-MSC to MM) or the other (from MM to BM-MSC) (see details in Table 2). Mitochondrial transfer between BM-MSCs and myeloma cells was not affected by any of these inhibitors because none of these compounds was able to reduce MT either from stromal cells to myeloma cells or vice versa in co-culture experiments. To understand the process of MT from BM-MSC to MMs, time-lapse imaging was carried out with a digital microscope system (high content screening). The images showed that within 1 h of the establishment of the co-cultures, myeloma cells tightly adhered to BM-MSCs, and some MMs contained stromal cell-derived mitochondria (Figure 7D and Supplementary Figure S5). Since 1 h is likely not sufficient for TNT formation, stabilization, and mitochondrial transfer through these membrane structures, we hypothesized that MT from BM-MSCs to MMs must use another transfer mechanism as well as that from MMs to BM-MSCs. The other possible mechanism of mitochondrial transfer from BM-MSCs to MMs may be cell-projection pumping: a mechanism possibly supported by a recent publication, where hydrodynamic cytoplasmic transfer and organelle transfer occurred between human fibroblasts and malignant cells via this pathway [34]. In order to confirm this hypothesis, 3D analysis of co-cultures with confocal scanning microscopy was carried out using transgenic BM-MSCs expressing the AcGFP1 fluorescence protein specifically tagged to the mitochondria.

These BM-MSCs were modified with a lentiviral transfection system (Supplementary Figure S7). As shown in Figure 7E, the MT occurred at the tight adhesion areas between the stromal cell and the MMs indicated by red arrows. In the case of the MM cells indicated by red arrows, it can be seen how the membrane protrusions of the myeloma cell surround the long projection of the stromal cell where the BM-MSC-derived mitochondria enter the MM cell through these cytoplasmic protrusions. It was also possible to observe how the apical surface of the MM cell attaches to the projection of BM-MSC where the mitochondrial transfer appears towards the malignant cell.

In the final experiments, to exclude any possible technical problem with mitochondrial staining method, MT was analyzed on one selected autologous myeloma cell culture modified with the foregoing lentiviral transfection system. Primary BM-MSCs or MMs were transduced with lentiviral particles delivering sequences that expressed mitochondria-targeted fluorescent protein tags (AcGFP1), and then these cells were co-cultured with control recipient MMs or BM-MSCs, respectively. Similar tendencies of MT were detected in the transfer in both directions in the presence of chemotherapeutic drugs, therapeutical antibodies, and cytochalasin D to those obtained with Mitotracker Red FM (Supplementary Figure S6). These results validated our data regarding TNT and MT analysis.

## 4. Discussion

Recent studies have supported that one of the important mechanisms by which tumor cells are able to overcome the toxic effect of therapeutical cell-killing drugs is mitochondrial transport between stromal cells and tumor cells. As it has been shown, mitochondrial transfer is not unidirectional [7,8,9,10,13,21]. On one hand, damaged mitochondria released from dying tumor cells or from distressed adjacent cells can be incorporated by mesenchymal stromal cells. On the other hand, MSCs can promote the initiation of reparative processes in suffering cells by transferring functional mitochondria [35,36,37,38,39]. Although MT between tumor and stromal cells and its consequences has been intensively studied in a plethora of hematological tumors [7,8,9,10,11,20,21,40], we focused on studying MT between myeloma cells and BM-MSCs. Uniquely, primary myeloma and autologous stromal cells from patients suffering multiple myeloma were used in this work instead of cell lines. Mitochondrial transfer was examined in the presence of drugs and antibodies used for MM therapy.

Therapeutic antibodies Daratumumab, Isatuximab (anti-CD38), and Elotuzumab (anti-CD319/SLAMF7) were not toxic to MMs, BM-MSCs, or their co-cultures. According to this finding, none of these antibodies reduced mitochondrial transfer between MMs and BM-MSCs, except Daratumumab, which significantly inhibited the MT from MMs to BM-MSCs; importantly, it did not block MT from BM-MSC to MMs, which result is similar to cytochalasin D. 

Marlein et al. argued [11] that CD38 antibody treatment resulted in significantly reduced MT. These inconsistencies are explained by the various experimental differences. We used primary, autologous MMs, and BM-MSCs obtained from MM patients, while myeloma cell lines were used in the cited publication. On the other hand, we used anti-CD38 therapeutic antibodies regularly applied in the clinical practice, while Marlein and colleagues worked with an unknown type of anti-CD38 antibody, which may differ from Daratumumab or Isatuximab regarding the epitope specificity and characteristics. 

Therapeutical drugs, the proteasome inhibitor carfilzomib, the BCL2 inhibitor venetoclax, and the HDAC inhibitor Na-valproate were highly toxic to primary MMs but not to BM-MSCs. In the co-cultures of MMs and BM-MSCs, the stromal cells reduced the death of MMs, and, in parallel, the amount of BM-MSC-derived mitochondria increased in the surviving MMs. The transfer of functional mitochondria was accompanied with a rise in ATP level and a decrease in mitochondrial superoxide level in MMs.

The opposite direction, MT from MMs to BM-MSCs, was also analyzed. Long-term (48 h) co-culture of MMs and BM-MSCs in the presence of the used drugs resulted in a drug concentration-dependent increase in MT from MMs to BM-MSCs. The correlation between the increasing drug concentration, the number of dead MMs, and the level of incorporation of MM cell-derived mitochondria by BM-MSCs was also established. The more the MMs died, the more MM-mitochondria were adopted by BM-MSC. Accordingly, incorporation of damaged MM cell-derived mitochondria by BM-MSCs did not result in changes in ATP level, but mitochondrial superoxide levels in stomal cells were elevated.

An increasing number of recent studies show that oxidative phosphorylation activity is elevated in several tumor types including lymphomas and leukemias, the high OXPHOS subtype of melanoma, pancreatic ductal adenocarcinoma, and endometrial or ovarian carcinoma [16,17,18,41]. Moreover, conventional chemotherapeutic drugs increase the mitochondrial transfer from stromal cells, and recipient tumor cells consistently show increased OXPHOS activity and ATP production and improved proliferative and migratory properties [8,12,39,42,43,44]. As the inhibition of oxidative phosphorylation is an emerging target in cancer therapy [16,41,45,46] and the simultaneous inhibition of oxidative phosphorylation enhances the effect of antitumor agents [13], co-targeting MMs with chemotherapeutic drugs and the OXPHOS metabolism inhibitor could be an effective adjuvant strategy in multiple myeloma to influence BM-MSC support and the critical metabolic function of MMs. Therefore, we used the OXPHOS inhibitor metformin in combination with carfilzomib, venetoclax, and Na-valproate on MM monocultures or MM–BM-MSC co-cultures. Although metformin did not influence the mitochondrial transfer between BM-MSCs and MMs, we showed that even the non-toxic dose of metformin significantly reduced MM cell viability in monoculture when used together with a non-toxic dose of chemotherapeutic drugs. Moreover, BM-MSCs in the co-cultures could not significantly influence MM cell survival in the presence of these drug combinations despite the bidirectional MT-stimulating effect of carfilzomib, venetoclax, and Na-valproate.

The mechanism of mitochondria exchange between tumor and stromal cells has been recently extensively studied [38,47]. Most of the studies state that TNTs are responsible for these events. Hence, we analyzed the role of tunneling nanotubes in the mechanism of mitochondrial transfer. Confocal microscopic images of live cell cultures showed unambiguous TNT formation between MMs and BM-MSCs; however, the TNTs exclusively seemed to stem from MMs. The widely used inhibitor of TNTs, cytochalasin D, which inhibits actin polymerization, was only able to block MT between myeloma cells and bone-marrow stromal cells. Cytochalasin D significantly inhibited MT from MMs to BM-MSCs, as expected, and these results are in accordance with previous findings [7,10,11]. In contrast, MT from BM-MSCs to MMs was clearly different from that from MMs to BM-MSCs. This inhibitor was not able to block MT from BM-MSCs to MMs indicating that mitochondrial incorporation by myeloma cells occurred on another pathway. Cytochalasin D slightly increased MT of this direction, supporting that the inhibition of actin polymerization upregulated MMs’ mitochondrial incorporation. During high-content screening studies, the analysis of the time-lapse records revealed that some myeloma cells were positive for BM-MSC-derived mitochondria as early as 1 h after co-culture establishment, which excludes the role of TNTs, since this short time was certainly not sufficient for TNT formation, stabilization, and mitochondrial transfer through these structures. The myeloma cells, which incorporated BM-MSC-derived mitochondria showed close adhesion to the stromal cell, and MM cell protrusions changing rapidly in position and shape appeared on the time-lapse records.

Our results disagree with the only study that has been published to date about mitochondrial transfer between BM-MSCs and multiple myeloma cells [11]. They argue that myeloma cells acquired stromal cell-derived mitochondria via TNTs; therefore, inhibition of actin polymerization by cytochalasin D blocked this process; they also state an exclusive role of TNTs in the mitochondrial transfer. To resolve this discrepancy, we validated our results using transgenic BM-MSCs expressing the mitochondrial-targeted AcGFP1 fluorescence protein in comparison to those of Mitotracker Red FM-labeled BM-MSC to exclude an effect of cytochalasin D other than the inhibition of actin polymerization and to exclude any possible technical problem with mitochondrial staining method. This experiment showed that the results obtained with the inhibitor cytochalasin were comparable to those obtained with transgenic cells. As cytochalasin D inhibited MT from MM to BM-MSC, while, contrarily, it elevated that from BM-MSC to MM, we suggest that MMs generate TNTs for mitochondrial transfer for both directions, but in the presence of cytochalasin D, MMs use another transfer mechanism for MT. We suggest a possible efficient alternative pathway; hydrodynamic cytoplasmic transfer resulting in mitochondrial transfer was recently published [34]. This mechanism occurred via cell-projection pumping between malignant cells and human fibroblasts. Although TNTs were detected, the intercellular transfer did not occur via TNTs. Instead, fine and often branching cell projections were involved, although direct visual resolution was rendered impossible due to their size and rapid movement. In accordance with this finding, we found a similar mechanism after the 3D analysis of PFA-fixed co-cultures. We used transgenic BM-MSCs expressing mitochondrial-targeted AcGFP1 fluorescence protein and MMs labeled with DiI membrane-labeling dye. According to the 3D images, the MT occurred at the tight adhesion areas between the stromal cell and the MMs. It is clearly seen how the membrane protrusions of the myeloma cell surround the long projection of the stromal cell or the apical surface of MM cells attached to the projection of BM-MSC where the mitochondria move towards the malignant cell.

**Figure 8:**
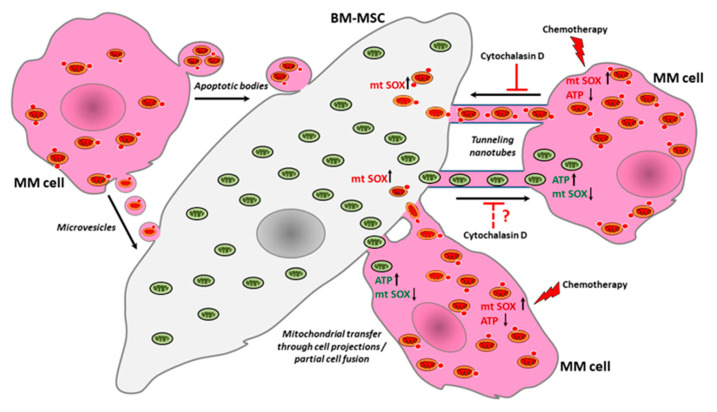
Mechanisms of bidirectional mitochondrial transfer between bone marrow stromal cells and myeloma cells. Bidirectional mitochondrial transfer occurs between autologous primary BM-MSCs and myeloma cells through MM cell-derived tunneling nanotubes. By inhibiting TNT formation by cytochalasin D, mitochondrial transfer is significantly reduced from MMs to BM-MSCs, but the incorporation of BM-MSC-derived mitochondria by myeloma cells continues through MM cell-derived cell projections after the fusion of cell membranes. MMs are unable to incorporate BM-MSC-derived MVs and hence mitochondria. In contrast, BM-MSCs incorporate large amounts of MM cell-derived mitochondria as a consequence of concentrated MV treatment, but this phenomenon is not decisive at physiological cell ratios in co-cultures. The incorporation of MM cell-derived mitochondria by BM-MSCs also occurs via endocytosis of MMs undergoing an early apoptotic phase or via the endocytosis of MM cell-derived apoptotic bodies induced by drug cytotoxicity. After chemotherapeutic drug treatment, independently from the type and mechanism of action of the drugs, BM-MSCs reduce the mortality of MMs, and, in parallel, the amount of BM-MSC-derived mitochondria increase in the surviving MMs. Transfer of functional mitochondria is accompanied with rise in ATP level and decrease in mitochondrial superoxide level in MMs. In parallel, there is also an increase in drug concentration-dependent mitochondrial transfer from MMs to BM-MSCs, increasing the level of free radicals in the stromal cells.

Our theory for the possible mechanisms of mitochondrial exchange between MMs and BM-MCSs is summarized in Figure 8.

## 5. Conclusions

In this study, we have highlighted that primary myeloma cells respond to increasing levels of chemotherapeutic drugs, such as the proteasome inhibitor carfilzomib, the BCL2 inhibitor venetoclax, and the HDAC inhibitor Na-valproate, with increasing acquisition of mitochondria from BM-MSCs whereupon the survival and ATP level increase, while mitochondrial superoxide levels decrease in myeloma cells. These changes and the elevation of superoxide level in stromal cells are proportional to the amount of incorporated mitochondria derived from the other cell type and to the concentration of the used drug, but independent from the type and mechanism of action of the medicine. Although the inhibition of mitochondrial transfer is limited between BM-MSCs and myeloma cells, and even therapeutic antibodies are unable to moderate this process in vitro, the supportive effect of stromal cells can be effectively avoided by influencing the tumor metabolism using the OXPHOS inhibitor metformin and chemotherapeutic drugs together. This effective adjuvant strategy and the contribution to knowledge about the relationships between bidirectional mitochondrial transfer and chemoresistance in multiple myeloma may have practical implications for both physicians and researchers involved in the therapy of multiple myeloma.

## Figures and Tables

**Table 1:** Multiple myeloma patients with excellent in vitro growth potential of neoplastic plasma cells used in this study.

Sample ID	Age	Sex	Ig Isotype	Primary Genetic Alteration	Newly Diagnosed/Relapsed
#108	83	Female	IgA lambda	t(4;14)	Relapsed
#118	70	Male	IgG kappa	Hyperdiploidy	Newly diagnosed
#123	85	Female	IgG kappa	t(4;14)	Relapsed
#126	74	Female	IgG lambda	Hyperdiploidy	Relapsed
#128	43	Male	Kappa light chain	Hyperdiploidy	Newly diagnosed
#130	53	Male	IgG kappa	Hyperdiploidy	Newly diagnosed
#132	56	Male	IgA kappa	t(4;14)	Newly diagnosed
#165	77	Female	IgA kappa	t(11;14)	Newly diagnosed
#178	77	Male	IgG kappa	Hyperdiploidy	Relapsed
#179	50	Female	Kappa light chain	t(11;14)	Newly diagnosed

**Table 2:** Therapeutic antibodies, chemotherapeutic drugs, and inhibitors used in this study.

Chemotherapeutic Drugs/Therapeutic Antibodies	Stock Solution Diluent/Concentration Range Tested	Manufacturer	In Vitro Mechanism of Action in MM—BM-MSC Co-Cultures	Assay/Subject of Investigation
Carfilzomib (Kyprolis)	DMSO/ 0–100 nM	Amgen Inc.	Proteasome inhibitor	Tested in cytotoxicity assay and investigation of the effect on mitochondrial transfer between MM cells and BM-stromal cells.
Venetoclax (HY-15531)	DMSO/ 0–50 µM	MedChem Express LLC	Bcl-2 inhibitor	
Sodium-valproate (Depakine)	Water/ 0–50 mM	Sanofi S.A.	Histone deacetylase (HDAC) inhibitor	
TIC10 (ONC201)	DMSO/ 0–1000 µM	MedChem Express LLC	Induces mitochondrial damage; indirectly inhibits mitochondrial respiration [22]	
Daratumumab—anti-CD38 mAB (Darzalex)	Solution for infusion/ 0–100 µg/mL	Janssen Biotech, Inc.	CD38 internalization and subsequent loss of adhesion to BM-MSCs [23]; cross-linking of tumor-bound monoclonal antibodies may induce programmed cell death [24,25]	
Isatuximab—anti CD38 mAB (Sarclisa)	Solution for infusion/ 0–100 µg/mL	Sanofi-Genzyme	Directly triggers MM cell death in the absence of cross-linking agents and independently of effector cells and Fc fragment binding to Fc receptors (caspase-dependent apoptotic pathway, lysosomal cell death pathway) [24,25,26,27]	
Elotuzumab—anti-CD319 mAB (Empliciti)	Solution for infusion/ 0–100 µg/mL	Bristol-Myers Squibb and AbbVie	Inhibits MM cell interaction with bone marrow stromal cells [28]	
**Inhibitors**	**Stock solution diluent/concentration range tested**	**Manufacturer**	**In vitro mechanism of action in MM–BM-MSC co-cultures**	**Assay/subject of investigation**
Dynasore	DMSO/ 0–100 µM	Merck KGaA	Endocytosis inhibitor	Investigation of the inhibitory effect on mitochondrial transfer alone or in the presence of carfilzomib, venetoclax, or na-valproate.
18α-Glycyrrhetinic acid	DMSO/ 0–100 µM	Merck KGaA	Gap junction blocker	
EIPA	DMSO/ 0–100 µM	MedChemExpress LLC	Macropinocytosis inhibitor	
Cytochalasin D	DMSO/ 0–10 µM	Merck KGaA	Actin polymerization inhibitor; abolishes TNT formation	
Colcemide	HBSS/ 0–10 µM	Thermo Fisher Scientific	Tubulin polymerization inhibitor; abolishes TNT formation	
Defibrotide (Defitelio)	Solution for infusion/ 0–100 µg/ml	Gentium S.r.l.	Inhibits MM cell adhesion with BM-MSCs [29]	
Metformin	HBSS/ 0–150 mM	Merck KGaA	OXPHOS inhibitor; interferes with TNT development [4,13,30]	

## Data Availability

The data are contained within the article and Supplementary Materials.

## Supplementary Materials

The following are available online at https://www.mdpi.com/article/10.3390/cancers13143461/s1. Figure S1: Detailed presentation of the experimental settings and assay setups of this study, Figure S2: Cytotoxic effect of therapeutic antibodies on BM-MSC or MM monocultures or BM-MSC–MM co-cultures, Figure S3: Mitochondrial transfer between BM-MSCs and myeloma cells in transwell assays, Figure S4: Microvesicle-mediated mitochondrial transfer between BM-MSCs and myeloma cells, Figure S5: Time-lapse images of mitochondrial transfer from bone marrow stromal cells to MM cells, Figure S6: Validating the mitochondrial transfer analysis applying transgenic BM-MSCs or MMs expressing mitochondrial-targeted AcGFP1 fluorescence protein, Figure S7: Lentiviral transduction of BM-MSCs or MM cells with pCT-Mito-GFP lentiviral particles, Figure S8: Illustration of gating strategies for FACS analysis of mitochondrial transfer.
