# Supplementary material for: Stromal Cells Serve Drug Resistance for Multiple Myeloma via Mitochondrial Transfer: A Study on Primary Myeloma and Stromal Cells
Source: Cancers (Basel). 2021 Jul 10;13(14):3461. doi: 10.3390/cancers13143461 (PMC8307863; doi:10.3390/cancers13143461)
Supplement: Supplementary file 1 [file cancers-13-03461-s001.zip › cancers-1255801-supplementary.pdf]

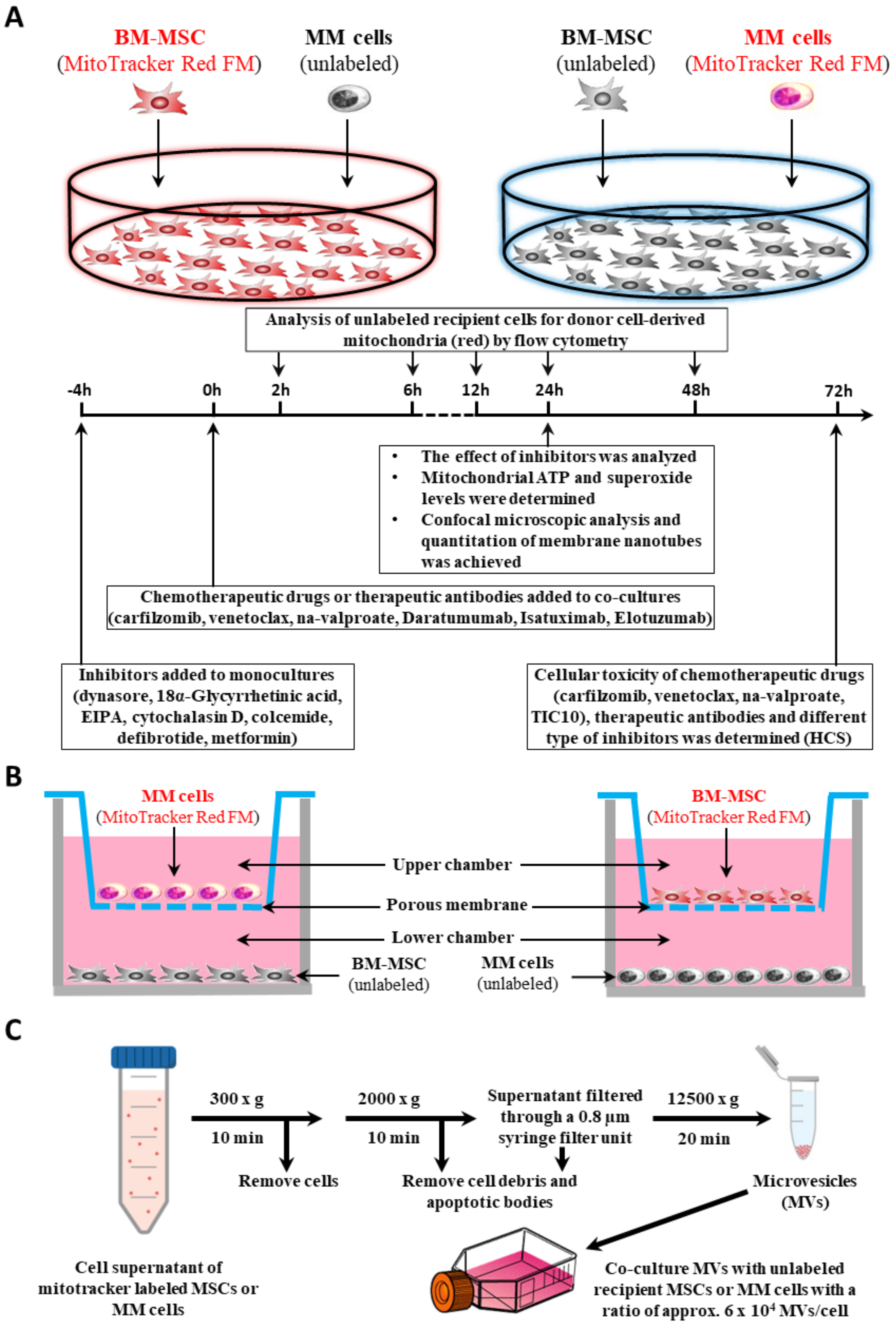

Supplementary Figure S1. The detailed presentation of the experimental settings and assay setups of this study. (A) Mitochodrial Transfer Assay. **(B)** Transwell assay. **(C)** Microvesicle (MV) assay

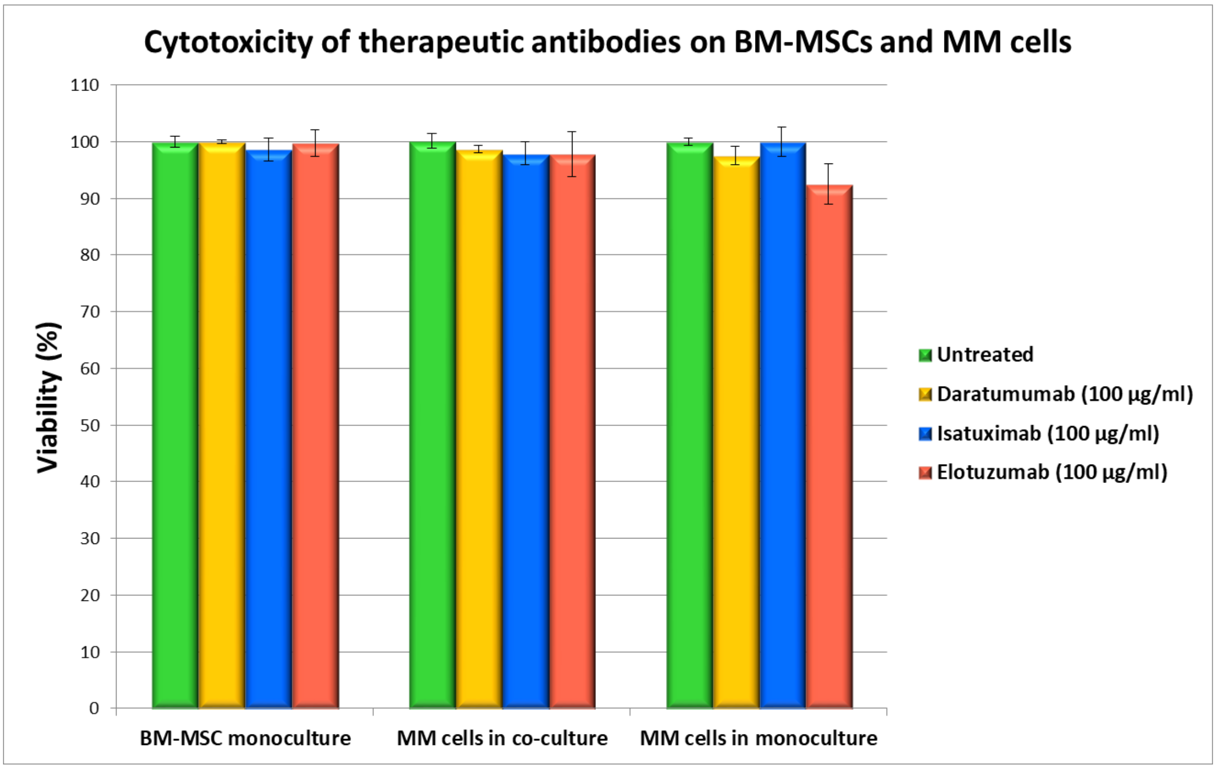

Supplementary Figure S2. Cytotoxic effect of therapeutic antibodies to BM-MSC or MM monocultures or BM-MSC – MM co-cultures. Monoclonal antibodies Daratumumab, Isatuximab and Elotuzumab were used at a concentration of 100 µg/ml, which is the optimal plasma concentration that is already effective in patients receiving Daratumumab.

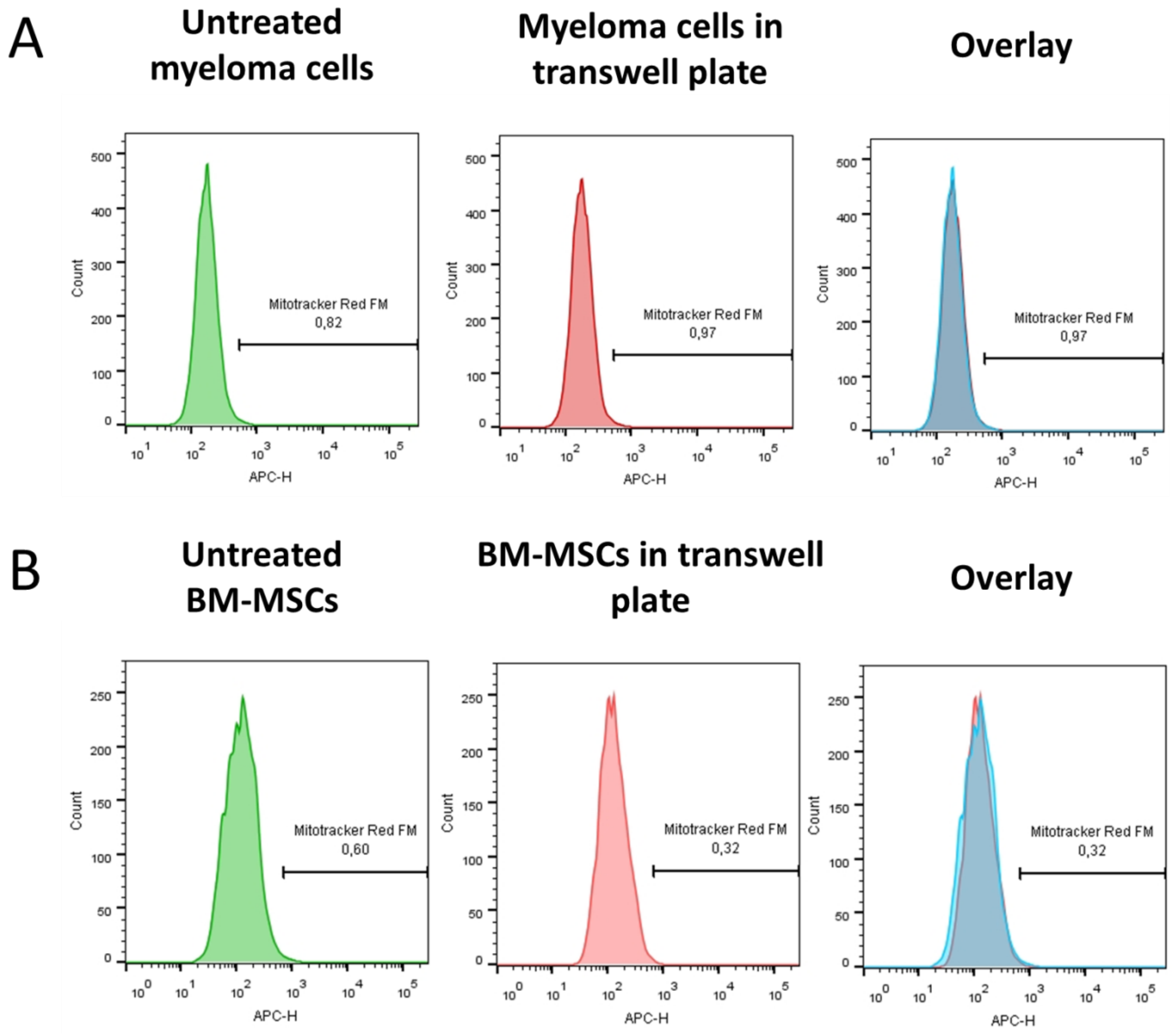

Supplementary Figure S3. Mitochondrial transfer between BM-MSCs and myeloma cells in transwell assays. The unlabeled BM-MSCs or MM cells were cultured in the lower chamber while the mitotracker labeled cells were cultured in the upper chamber for 24 hours before FACS analysis of the unlabeled recipient cells.

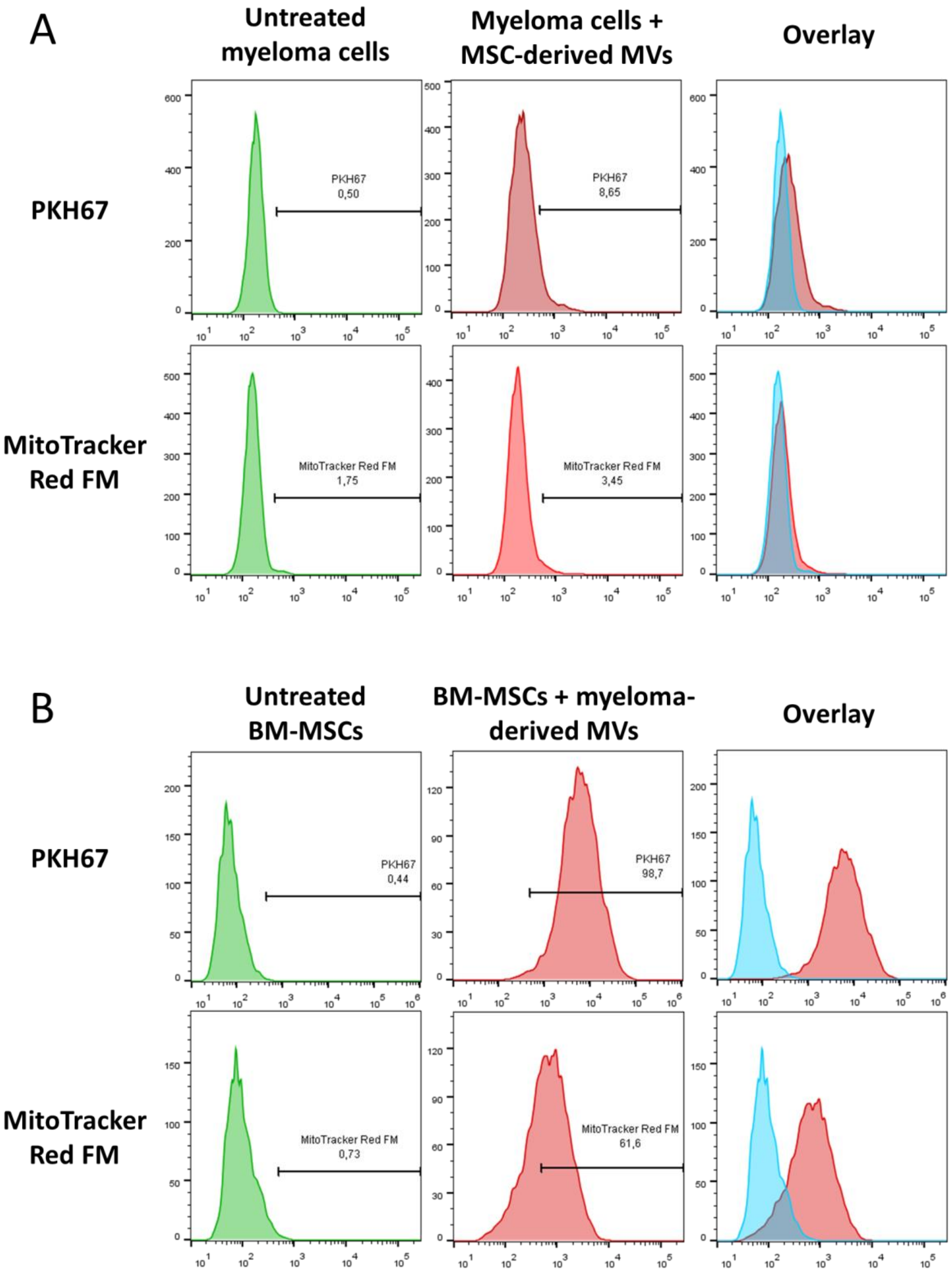

Supplementary Figure S4. Microvesicle-mediated mitochondrial transfer between BM-MSCs and myeloma cells. Microvesicles were labeled with PKH67 membrane labeling dye and Mitotracker Red FM (**A** and **B**). Myeloma cells or BM-MSCs were incubated with BM-MSC-derived MVs (**A**) or MM derived MVs (**B**), respectively and after co-incubating isolated microvesicles with cells for 24 hours, the samples were analyzed with cytofluorimetry.

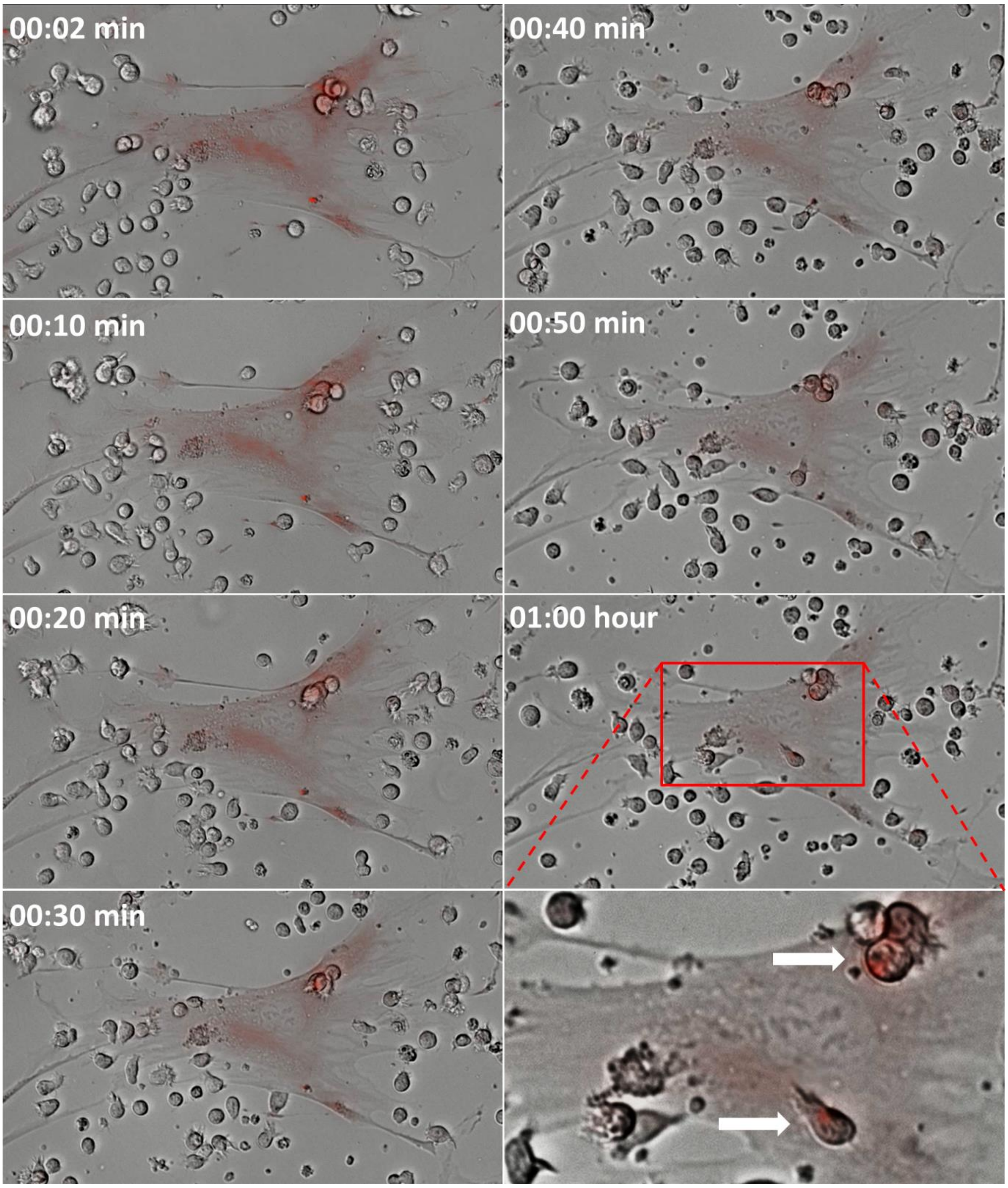

Supplementary Figure S5. Time-lapse images of mitochondrial transfer from bone marrow stromal cells to MM cells.

Time-lapse imaging of BM-MSC-MM co-culture was used to study mitochondrial transfer from BM-MSCs to myeloma cells. BM-MSCs were stained with Mitotracker Red FM while MM cells were unlabeled. White arrows show MSC derived mitochondria in MM cells.

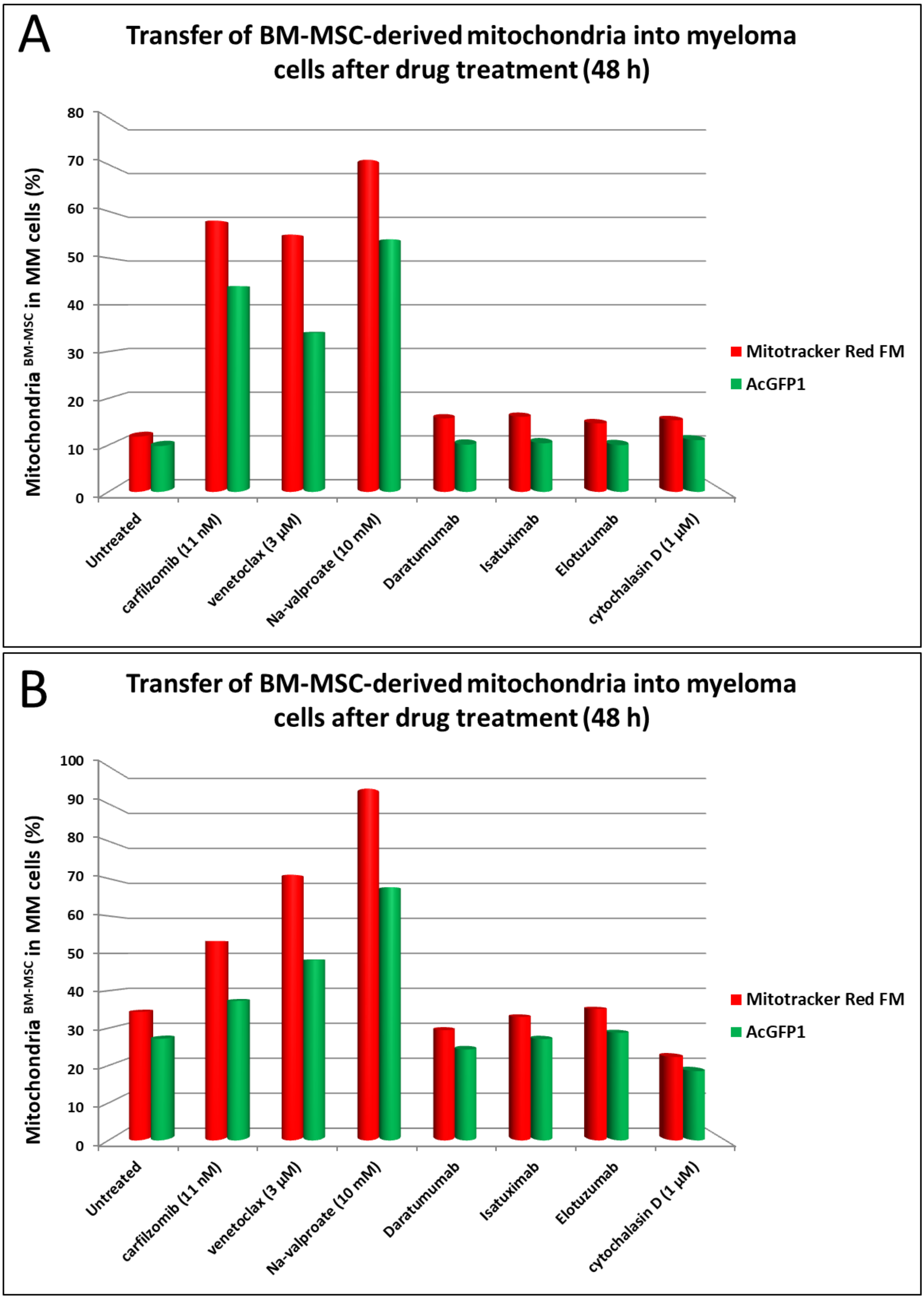

Supplementary Figure S6. Validating the mitochondrial transfer analysis applying transgenic BM-MSCs or MMs expressing mitochondrial-targeted AcGFP1 fluorescence protein. (A) The mitochondrial compartment of the donor BM-MSCs was either labeled with the MitoTracker Red FM dye, or due to prior lentiviral transduction these stromal cells

expressed mitochondrial-targeted AcGFP1 fluorescence protein. The BM-MSC-derived mitochondrial positivity of the recipient myeloma cells in co-cultures was analyzed with cytofluorimetry after 48 hours. **(B)** The mitochondrial compartment of the donor myeloma cells was either labeled with the MitoTracker Red FM dye, or due to prior lentiviral transduction these myeloma cells expressed mitochondrial-targeted AcGFP1 fluorescence protein. The MM cell-derived mitochondrial positivity of the recipient BM-MSCs in co-cultures was analyzed with cytofluorimetry after 48 hours. The co-cultures were treated with chemotherapeutic drugs (carfilzomib, venetoclax, Na-valproate), therapeutic antibodies (Daratumumab, Isatuximab, Elotuzumab) or cytochalasin D.

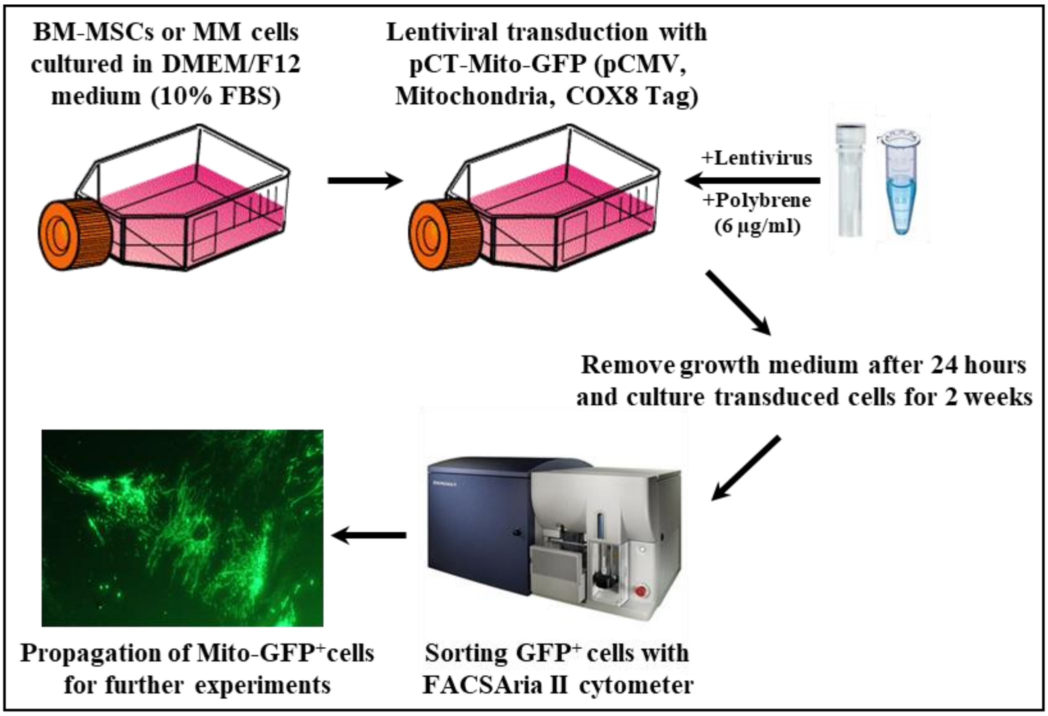

Supplementary Figure S7. Lentiviral transduction of BM-MSCs or MM cells with pCT-Mito-GFP lentiviral particles. Lentiviral particles were added to the growth medium of BM-MSCs or myeloma cells at a multiplicity of infection (MOI) of approx. 10 (BM-MSCs) or 25 (MM cells). Polybrene was added to the medium as well in a final concentration of 6 µg/ml. After 24 hours, culture medium was removed and cells were cultured for 2 weeks. GFP^+ cells were sorted with FACSAria II cytometer and cultured for further experiments.

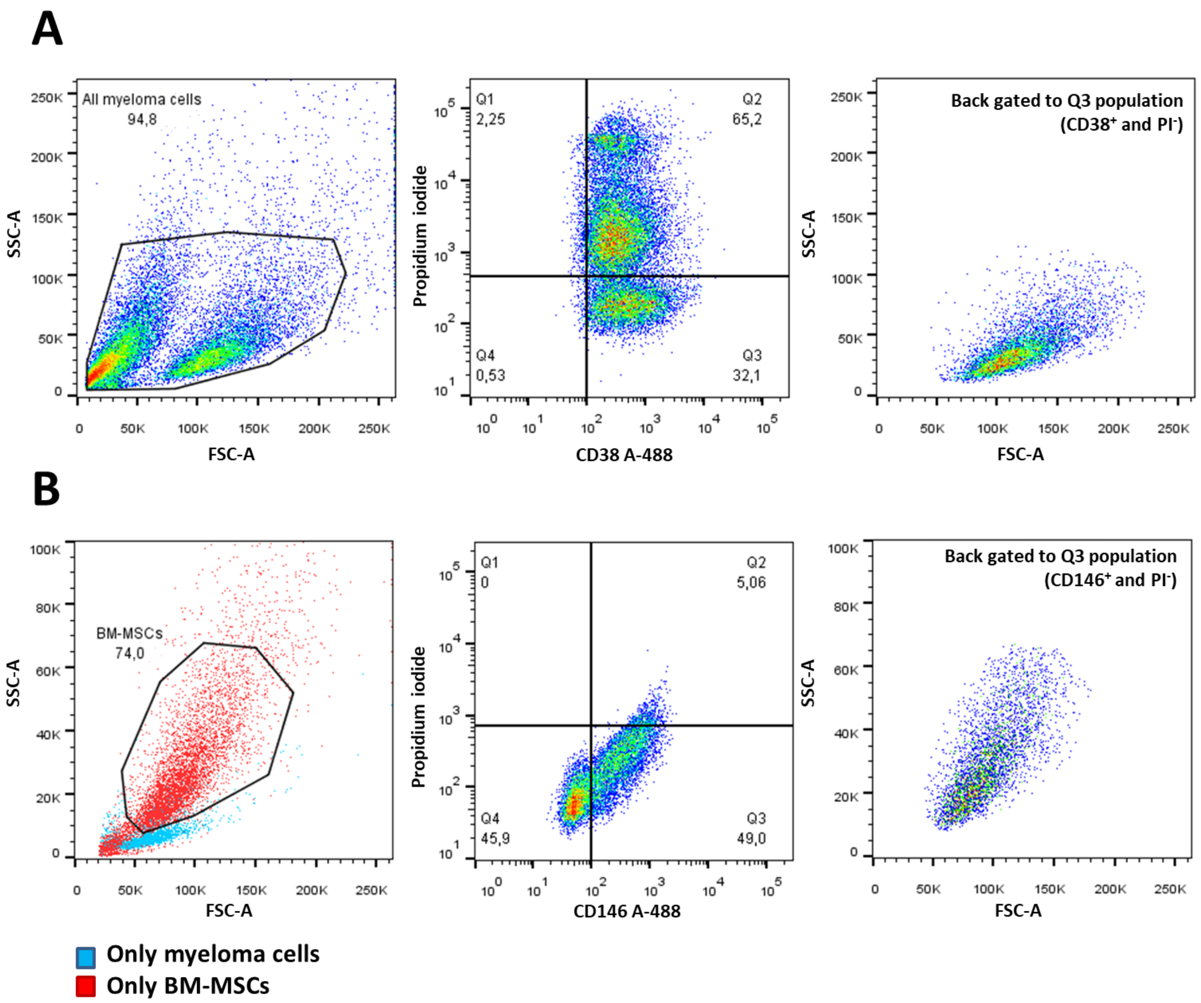

Supplementary Figure S8. Illustration of gating strategies for FACS analysis of mitochondrial transfer. The gating strategy to measure MMs in co-cultures is shown in **Panel A**. The living and dead myeloma cells can be easily distinguished according to their different distribution on the FSC vs. SSC dot-plot, but for the MitoTracker analysis the $CD38^+$ and PI^- population (Q3) was used. All myeloma cells derived from different donors were highly positive for CD38. The gating strategy to measure BM-MSCs in co-cultures is shown in **Panel B**. BM-MSCs can be easily distinguished from MMs according to their different distribution on the FSC vs. SSC dot-plot (large difference in size), but for the MitoTracker analysis the $CD146^+$ and PI^- population (Q3) was used. Although 50-80% of the BM-MSCs were positive for CD146 on average, we used consistently the $CD146^+$ population for further analysis in order to avoid measuring myeloma cells in addition to stromal cells.
